# Methylome profiling of cell-free DNA during the early life course in (un)complicated pregnancies using MeD-seq: Protocol for a cohort study embedded in the prospective Rotterdam periconception cohort

**DOI:** 10.1371/journal.pone.0310019

**Published:** 2025-01-09

**Authors:** Marjolein M. van Vliet, Sam Schoenmakers, Ruben G. Boers, Lotte E. van der Meeren, Joost Gribnau, Régine P. M. Steegers-Theunissen

**Affiliations:** 1 Department of Obstetrics and Gynaecology, Erasmus Medical Centre Rotterdam, Rotterdam, The Netherlands; 2 Department of Developmental Biology, Erasmus Medical Centre Rotterdam, Rotterdam, The Netherlands; 3 Department of pathology, Erasmus Medical Centre Rotterdam, Rotterdam, The Netherlands; 4 Department of Pathology, Leiden University Medical Center, Leiden, The Netherlands; Universita degli Studi di Milano Facolta di Medicina e Chirurgia, ITALY

## Abstract

**Introduction:**

Placental DNA methylation differences have been associated with timing in gestation and pregnancy complications. Maternal cell-free DNA (cfDNA) partly originates from the placenta and could enable the minimally invasive study of placental DNA methylation dynamics. We will for the first time longitudinally investigate cfDNA methylation during pregnancy by using Methylated DNA Sequencing (MeD-seq), which is compatible with low cfDNA levels and has an extensive genome-wide coverage. We aim to investigate DNA methylation in placental tissues and cfDNA during different trimesters in uncomplicated pregnancies, and in pregnancies with placental-related complications, including preeclampsia and fetal growth restriction. Identified gestational-age and disease-specific differentially methylated regions (DMRs) could lead to numerous applications including biomarker development.

**Methods and analysis:**

Our study design involves three sub-studies. Sub-study 1 is a single-centre prospective, observational subcohort embedded within the Rotterdam Periconception cohort (Predict study). We will longitudinally collect maternal plasma in each trimester and during delivery, and sample postpartum placentas (n = 300). In sub-study 2, we will prospectively collect first and second trimester placental tissues (n = 10 per trimester). In sub-study 3 we will retrospectively collect plasma after non-invasive prenatal testing (NIPT) in an independent validation case-control cohort (n = 30–60). A methylation-dependent restriction enzyme (LpnPI) will be used to generate DNA fragments followed by sequencing on the Illumina NextSeq2000 platform. DMRs will be identified in placental tissues and cell types, and in cfDNA related to gestational-age or placental-related complications. (Paired) placental methylation profiles will be correlated to DMRs in cfDNA to aid tissue-of-origin analysis. We will establish a methylation score to predict associated diseases.

**Discussion:**

This study will provide insights in placental DNA methylation dynamics in health and disease, and could lead to clinical relevant biomarkers.

## Introduction

DNA methylation is an important epigenetic mechanism involved in the regulation of gene expression and plays a vital role in placental development and function [1–3]. The genome-wide distribution of DNA methylation in placental tissue is known to change with increasing gestational age [4–6] and we and others have shown changes in placental DNA methylation in postpartum placentas associated with major obstetric complications, including preeclampsia, intra-uterine growth restriction, and preterm birth [7–17]. Therefore, the study of placental DNA methylation potentially provides more insight in normal placental development and underlying pathophysiology of placental-related complications and can be used to develop both gestational age-specific as well as disease-specific biomarkers.

Previous DNA methylation studies mainly focussed on postpartum placental tissues. Placental methylation profiles of early and mid-gestation placentas are largely understudied due to the impossibility to non-invasively investigate placental tissue during (uncomplicated) pregnancy. However, a promising alternative to study placental DNA methylation is by use of blood plasma derived cell-free DNA (cfDNA) originating partly from turnover and spill of placental tissue into the maternal circulation. This is also referred to as cell-free fetal DNA, since placental and fetal tissue largely share the same DNA sequence. cfDNA is already widely used for non-invasive prenatal testing (NIPT) for chromosomal aneuploidies as part of prenatal screening [18].

Research into the epigenetic features of (fetal) cfDNA is suggested to allow for cfDNA applications beyond current practice [19, 20]. Thus far, studies investigating genome-wide methylation of cfDNA during pregnancy are scarce, but promising results have been reported recently indicating the feasibility of predicting the risk for preeclampsia by cfDNA methylome profiling at an earlier pregnancy stage [21–23]. Early identification of pregnancies at high-risk of developing preeclampsia is of the utmost importance to enable timely targeted preventative interventions such as the initation of low-dose aspirin and subsequently decrease maternal and fetal morbidity and mortality [24–26].

A previous developed ‘placental epigenetic clock’ showed the possibility to estimate gestational age using DNA methylation differences in placental tissues [27]. Although studies using cfDNA are limited, differences in methylation levels of cfDNA fragments between trimesters are reported [28]. This indicates the feasibility of identifying gestational age-specific methylation markers in cfDNA. This could increase our understanding of placental epigenetic programming during development, and could be used to assess gestational age using maternal blood. This could be relevant in circumstances where the widely used first-trimester ultrasound is not always available or when pregnancies are discovered in second or third trimester, when assessing gestational age using ultrasound is less reliable [29].

Specificity of cfDNA as a placental-specific marker is hampered by a background of cfDNA originating from maternal tissues. The placental-derived fraction in maternal cfDNA is about 10% at the end of the first trimester and increases with gestational age [30–32]. Since DNA methylation patterns are cell type specific [1], identification of methylation patterns in cfDNA specific for placental cell types will increase specificity of cfDNA as placental-specific marker.

Most techniques used to study DNA methylation are limited to a subset of cytosine-guanine (CpG) dinucleotides and rely on bisulfite conversion which substantially degrades DNA [33–35]. This challenges DNA methylation analyses of cfDNA which is generally only present in low amounts. We will therefore use the Methylated DNA sequencing (MeD-seq) technique. By omitting bisulfite conversion, MeD-seq is well compatible with low amounts of (cf)DNA as shown in previous studies performed using MeD-seq [36–39]. MeD-seq makes use of a methylation-dependent restriction enzyme (LpnPI) which activity is restricted by short fragment size. In contrast to other Methylation Dependent enzymes, which over-digest CpG dense regions of the genome, LpnPI does not have this problem and is able to generate DNA fragments from methylated CpG islands that are still large (around 32 bp) enough to be isolated, sequenced, and properly mapped. This limits the required sequencing depth while allowing for genome-wide methylation profiling of >50% of all potentially methylated CpGs, detecting >99% of all CpG islands and promoters, a more extensive coverage as compared to most other techniques [40, 41].

In summary, research gaps exist regarding dynamics of the placental DNA methylome over gestation and in the context of placental-related complications. Placental-originated cfDNA has the potential to facilitate minimally invasive placental methylome profiling during pregnancy and recent studies indicate the feasibility of its use in early preeclampsia prediction [22, 23]. Because of the extensive genome-wide coverage of the MeD-seq technology, we expect to identify additional differentially methylated regions (DMRs) related to normal placental development and placental-related complications that can improve the current use of cfDNA.

### Aims and objectives

We hypothesize that our study can identify several novel trimester-specific and disease-specific DMRs in placental tissues and (fetal) cfDNA. This could lead to numerous (pre)clinical applications including improved biomarker development for the prediction of obstetric complications. To this end, we aim to investigate genome-wide DNA methylation in both placental tissues and (fetal) cfDNA during different trimesters of gestation and in the context of placental-related complications in three different sub-studies, using the MeD-seq technology.

The study objectives include:

1. Identification of DNA methylation profiles in placental tissues and specific placental cell types in uncomplicated pregnancies in each trimester (**sub-study 1 and 2**);
  - Identify DMRs between trimesters and between different placental cell types
  - Use identified DNA methylation profiles as reference in tissue-of-origin analyses of DMRs in cfDNA
2. Identification of methylation profiles in cfDNA in uncomplicated pregnancies in each trimester and at delivery (**sub-study 1**);
  - Identify DMRs between trimesters and at birth in cfDNA
  - Investigate overlap of DMRs with DNA methylation marks in (paired) placental tissues
3. Identification of methylation profiles in cfDNA in pregnancies with obstetric complications including preeclampsia and fetal growth restriction in each trimester and at delivery (**sub-study 1 and 3**);
  - Identify disease-specific DMRs in each trimester and at birth in cfDNA from complicated pregnancies compared to uncomplicated pregnancies
  - Investigate overlap of DMRs with DNA methylation marks in (paired) placental tissues
  - Establish a methylation score able to predict disease-risk of associated complications based on top-ranked DMRs
  - Validate first trimester findings in an independent case-control cohort (**sub-study 3**)

Subsequently, underlying genetic pathways related to identified DMRs involved in normal placental development or disease development will be investigated to increase our understanding of epigenetic regulation during placental development.

## Methods and analysis

### Study design

**Fig 1:**
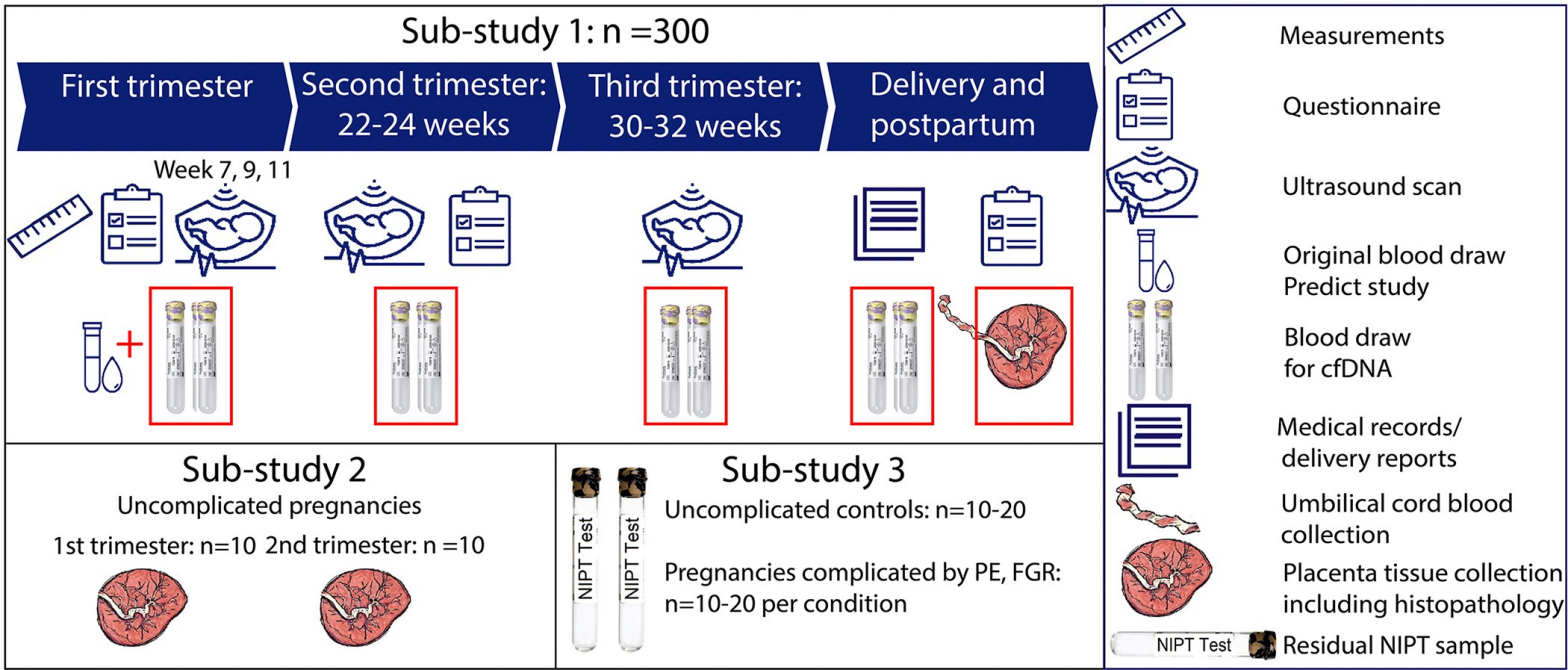
Overview of collected tissue materials and study procedures in the different sub-studies. Procedures in the current subcohort (sub-study 1) in addition to regular procedures in the Predict study during pregnancy are outlined in red.

Our study design involves three simultaneously conducted sub-studies (Fig 1). Sub-study 1 is a single-centre, prospective, observational sub-cohort study which is embedded in the Rotterdam Periconceptional cohort (Predict study) [42, 43] in which we longitudinally collect cfDNA in each trimester during pregnancy and during delivery, and we sample placental tissues postpartum. Inclusions for this subcohort started November 2^nd^ in 2022 and the total study duration is expected to take thirty months. Sub-study 2 is a cross-sectional study in which we prospectively collect placental tissues of uncomplicated pregnancies of the first and second trimester at an elective abortion clinic from July 10, 2023 onwards. Lastly, sub-study 3 is a retrospectively selected case-control study in which we will collect residual plasma samples previously collected for NIPT to validate our first trimester cfDNA findings in relation to obstetric complications in an independent cohort. The Standard Protocol Items: Recommendations for Interventional Trials (SPIRIT) checklist for this protocol can be found in S1 File.

### Ethics

Approval of all different parts of this protocol was obtained by the Medical Ethics Committee from the Erasmus MC, Rotterdam, The Netherlands. The Predict study was prospectively registered previously (NL4115 Dutch Trial Register). Approval for sub-study 1 which is embedded in the Predict study, was received in October 2022 (MEC-2004-0227, amendment A-0003). Approval for sub-study 2 version 1.1 was obtained in April 2023 (MEC-2023-0028) and for amendment A-0001. For sub-study 3 version 2.0 and amendment A-0001, approval was obtained in December 2023 (MEC-2022-0788). A copy of the approved protocols can be found in S2 File. Any study amendments will first be submitted for approval by the Medical Ethics Committee.

All samples collected within sub-study 1 and 3 are pseudo-anonymously coded with a unique study number. All samples within sub-study 2 are anonymously collected. The monitoring of the Predict study is followed for sub-study 1, which includes an independent monitor of the data monitoring committee of the Erasmus MC [42, 43]. The final trial dataset is accessible for the research team of the Predict study.

### Study population and recruitment

In sub-study 1, the same in-and exclusion criteria are followed as for the Predict study [42, 43]. All participants will be included at the outpatient clinic of the Erasmus MC, Rotterdam. Women of at least 18 years old with a singleton pregnancy who are <10+0 weeks pregnant are eligible for participation if they have sufficient understanding of the Dutch language and are willing to give written informed consent. Women pregnant after oocyte donation will be excluded. Written informed consent is asked by a research nurse at the Erasmus MC.

In sub-study 2, participants are included at a Dutch abortion clinic. Women of at least 18 years old with a singleton pregnancy with no known birth defects and a gestational age between 9+0–12+0 or 16+0–17+6 are eligible for participation. To ensure anonymity of participants, written informed consent is asked by a local nurse or medical doctor not involved in this study. Written informed consent is asked after choosing for elective, surgical abortion for social indications. Potential participants are excluded if they are infected with HIV, hepatitis B or, other blood borne infectious diseases.

Lastly, in sub-study 3, women will retrospectively be selected if they gave written informed consent to use residual NIPT material for research purposes. Between April 2017 and April 2023, all pregnant women in the Netherlands were offered NIPT on a trial basis (TRIDENT-2 study) [44, 45]. Women who previously participated in the Predict study and/or delivered at the Erasmus MC and who developed preeclampsia or fetal growth restriction will be matched with women who had uncomplicated pregnancies.

Preeclampsia will be defined according to the ISSHP guidelines [46]. Preeclampsia will be diagnosed if there is new-onset hypertension (≥140/90 mmHg) after 20 weeks of gestation, combined with at least one of the following new-onset complications: proteinuria; maternal end-organ dysfunction including thrombocytopenia, elevated liver enzymes, acute kidney injury, pulmonary edema, or, neurological symptoms (such as eclampsia, severe headaches, visual scotomata, stroke); or uteroplacental dysfunction (such as fetal growth restriction, abnormal umbilical artery Doppler wave form analysis, placental abruption). In women with chronic hypertension, superimposed preeclampsia will be diagnosed after development of new proteinuria, one of the above mentioned maternal organ dysfunctions, or signs of uteroplacental dysfunction. We will further distinguish early onset (<34 weeks of gestation) and late onset (≥34 weeks of gestation) preeclampsia [46].

### Study procedures: Sub-study 1

For women participating in sub-study 1, there will be multiple study visits including different measurements and procedures. The design and logistics of the Predict study has extensively been described earlier [42, 43]. Fig 1 gives an overview of visits and procedures as part of the Predict study and adaptations to the protocol for women in our subcohort.

In short, sub-study 1 consist of the following measurements and procedures:

### Anthropometrics

Height, weight, body circumferences, and blood pressure will be measured at study entry by a research nurse.

### Questionnaires

In the first trimester, a food frequency questionnaire and a general questionnaire focusing on lifestyle and general (health) information including geographical background will be filled out. At 24 weeks of gestation and after delivery, general questionnaires will be filled out focusing on lifestyle and pregnancy outcomes.

### Ultrasound scans

At 7, 9, and 11 weeks of gestation, 3D ultrasounds scans are performed using a GE Voluson E10 Expert system with a 6–12 megahertz transvaginal probe and 4D View software (General Electric Medical Systems, Zipf, Austria) focusing on embryonic and placental (vascular) development. At 22–24 weeks and 30–32 weeks of gestation, fetal growth will be assessed by fetal biometry and ultrasound Dopplers of the umbilical and uterine arteries and the middle cerebral artery will be performed.

### Blood samples

Blood samples will be collected in each trimester at the same day as the ultrasound. In addition to the standard Predict protocol, two CellSave preservative tubes (10mL each) are used to collect blood for subsequent cfDNA analyses at each time point. In the first trimester, blood is collected at 11 weeks of gestation which is comparable to timing of the national protocol for the NIPT [44]. If the delivery takes place at the Erasmus MC, blood will be collected after admission at the day of delivery before childbirth. Plasma will be isolated by two centrifugation steps (10 minutes at 1,711 g—2,000 g followed by 10 minutes 12,000 g—16,000 g) within 96 hours after blood collection and stored at −80°C before cfDNA isolation takes place.

### Postpartum tissues

After delivery, umbilical cord blood will be collected in one EDTA tube and a separator tube (both 10ml) and placentas will be formalin fixed. Histopathological examination of the placenta will be performed and formalin-fixed paraffin embedded (FFPE) blocks containing placental tissues are stored.

### Medical records

Medical records including delivery reports will be used to obtain detailed information regarding pregnancy outcomes, such as date of birth, birthweight, fetal sex, and the occurrence of obstetric complications such as preeclampsia.

#### Study procedures: Sub-study 2

In sub-study 2, participation of women will be anonymized and the only data collected is the gestational age at the time of elective abortion and the placental tissues. Participants will not undergo additional study procedures, since only leftover tissues will be collected. After the surgical procedure, placental tissues will be stored in formaldehyde solution 4%, buffered.

#### Study procedures: Sub-study 3

In sub-study 3 there will be no additional study procedures for participants, since only residual NIPT samples will be used.

#### Laser capture microdissection

**Fig 2:**
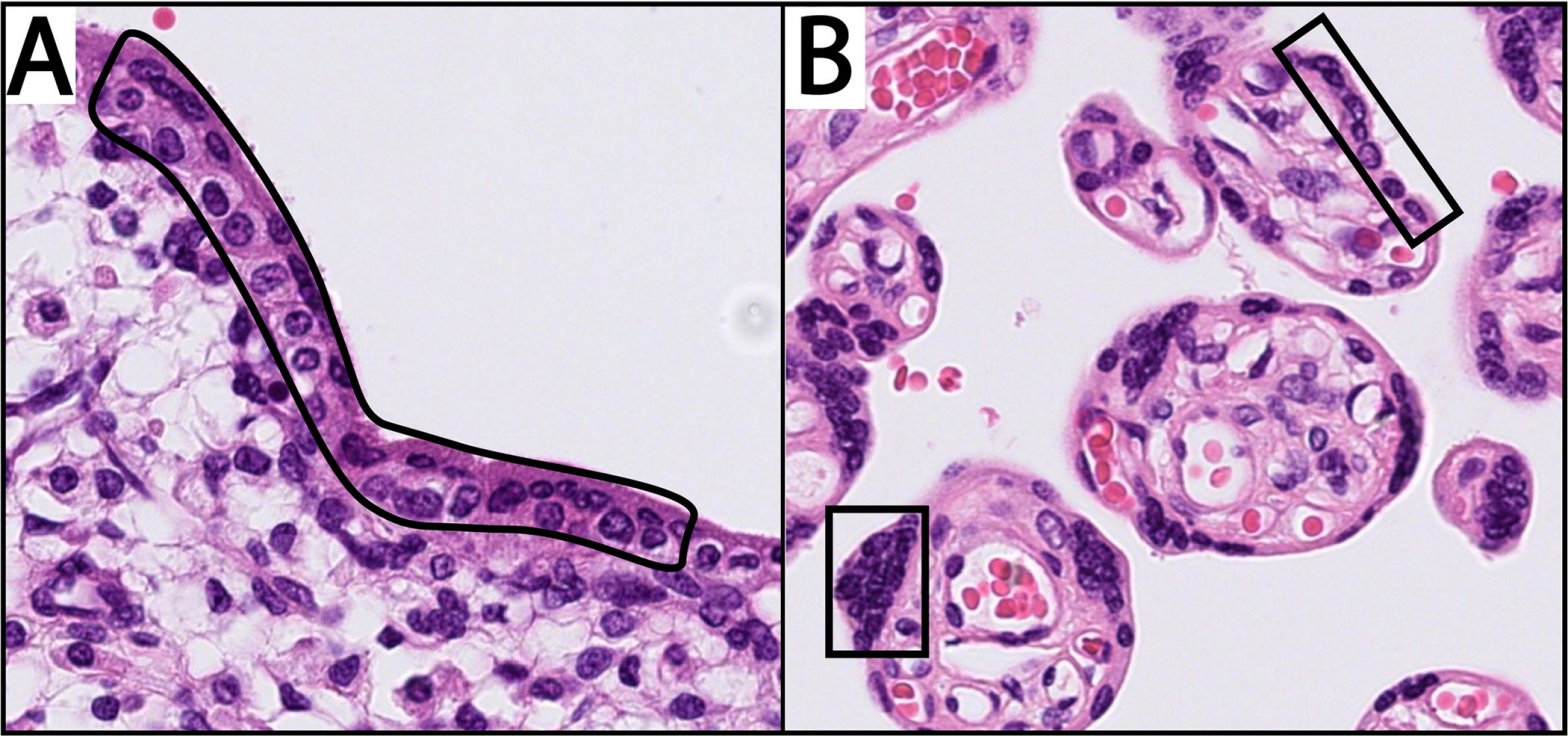
Part of placental cell types that will be separated by laser capture microdissection, Hematoxylin and Eosin (H&E) Stain, 40x magnification. A) First trimester placenta: outlined is the double layer of trophoblasts consisting of syncytiotrophoblast (outer layer) and cytotrophoblast (inner layer). B) Third trimester placenta: outlined are knob-shaped trophoblast (syncytial knots) (under, left) and the single trophoblast layer (up, middle).

DNA methylation patterns are cell type specific [1]. To be able to correlate DNA methylation profiles in placental tissues and cfDNA to specific placental cell types, we aim to obtain reference DNA methylation profiles for different placental cell types. This will increase specificity of cfDNA as placental-specific marker. An experienced technician and expert perinatal pathologist will perform laser capture microdissection (LCM) to separate different placental cell types, including extravillous trophoblasts and the double trophoblast layer consisting of syncytiotrophoblast (SCT) and cytotrophoblast (CTB) in first and second trimester, and the single trophoblast layer and knob-shaped trophoblasts (syncytial knots) in third trimester (Fig 2) [47]. A previous study showed compatibility of MeD-seq with samples isolated after LCM [48]. Cell types will be separated from 3 samples per trimester from uncomplicated pregnancies. First and second trimester samples are collected in sub-study 2 and third trimester samples are collected in sub-study 1 (Fig 1).

#### (cf)DNA isolation

Both bulk DNA isolation and cell type specific DNA isolation of LCM samples will be performed using the QIAamp DSP DNA FFPE Tissue kit according to the manufacturer protocol. All cfDNA will be isolated from 4 ml of plasma using the QIAamp Circulating Nucleic Acid Kit according to the manufacturer protocol.

### Sample size

Based on our previous experiments to generate reference profiles using MeD-seq, the number of reliable DMRs stabilizes when having a sample size of ±8–9 samples, as for example shown in [48]. Based on preliminary data, we also observed this stabilization in number of identified DMRs between cfDNA collected in the first trimester in sub-study 1 compared to cfDNA from non-pregnant controls (S1 Fig). This intended sample size is also supported by two recent cfDNA studies which identified robust methylation differences between women with preeclampsia and uncomplicated control pregnancies (n = 5–6 per group) [21, 23]. Therefore we aim to include 10 samples per time point for both cfDNA and direct placental tissues, and for both complicated and uncomplicated pregnancies.

In the Predict study, around 4% of pregnancies are complicated by preeclampsia and around 5% of children in this cohort is born with a birthweight <3th percentile. To include enough women who will develop above mentioned complications, we aim to prospectively include at least 300 pregnant women in sub-study 1.

In sub-study 2, we will collect 10 placentas in first and 10 placentas in second trimester and compare these with 10 third trimester placentas collected postpartum from uncomplicated pregnancies in sub-study 1 (total n = 30).

In sub-study 3, we will retrospectively collect samples from: 1. women who developed preeclampsia, distinguishing women with early onset as well as late onset preeclampsia, 2. women with fetal growth restriction and 3. uncomplicated control pregnancies (n = 10–20 per group).

### Data-analysis

MeD-seq assays will be performed as previously described [40, 41]. In short, genomic DNA and plasma-derived cfDNA are digested with the methylation-dependent restriction enzyme LpnPI (New England Biolabs, Ipswich, MA) generating 32 bp DNA fragments containing the methylated CpG in the middle, followed by sequencing on the Illumina NextSeq2000 platform. Custom Python scripts will be used to process the acquired DNA methylation profiles for the different samples using a previously established pipeline [40, 41]. Raw data files (fastq) will be filtered to be able to distinguish methylation data from background data and will be mapped to the human genome (hg38) using bowtie. We will generate regional (promoter, gene body, and CpG Island regions) methylation scores. For the genome-wide analysis, a sliding window technique will be used to detect DMRs and the Chi-squared test will be used for statistical testing with a Bonferroni correction. A Bonferroni adjusted p-value ≤0.05 will be considered significant. After DMRs are determined, Z-score transformation of the read count data will be applied for unsupervised hierarchical clustering analyses.

Our primary outcomes are DMRs in placental tissues and specific placental cell types between trimesters, DMRs in cfDNA in uncomplicated pregnancies between trimesters and at delivery, and DMRs in cfDNA in pregnancies with obstetric complications including fetal growth restriction and preeclampsia in each trimester and at delivery. (Paired) cfDNA and placental DMRs will be correlated to investigate the placental-origin of identified DMRs in cfDNA.

**Fig 3:**
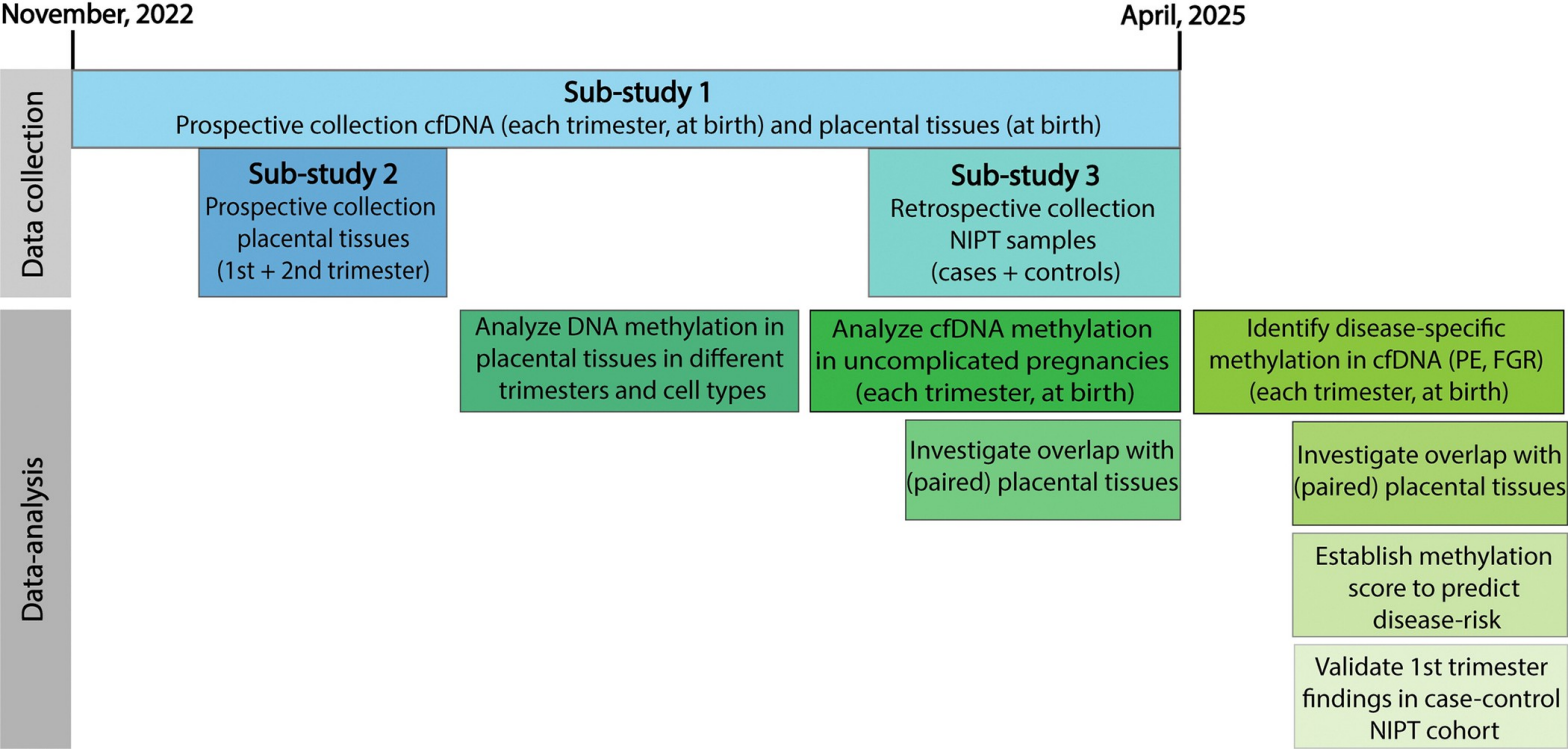
Timeline of data collection in the different sub-studies and data-analysis.

The intended timeline of data collection and data-analysis is depicted in Fig 3. We start our data-analysis with the characterisation of DNA methylation profiles in bulk placental DNA and in different placental cell types in each trimester to create a reference placental methylation map. Used placental tissues are collected in sub-study 1 and sub-study 2 from uncomplicated pregnancies. DMRs will be identified between placental cell types and between trimesters. Next, DMRs will be identified between cfDNA collected from uncomplicated pregnancies at the four different time points in sub-study 1. Direct comparison between placental tissues and paired cfDNA collected at delivery will allow for accurate identification of the placental contribution to cfDNA and overlap with the established reference placental DNA methylation map will be investigated.

Lastly, DMRs will be identified in cfDNA between (retrospectively collected) uncomplicated pregnancies and pregnancies complicated by major obstetric complications, including preeclampsia or fetal growth restriction. Sensitivity analysis will be performed for women with early or late onset preeclampsia where possible. In sub-study 1, DMRs will be investigated for each trimester and cases and controls will be matched based on maternal age, maternal BMI, parity, mode of conception, fetal sex, ethnicity, presence of gestational diabetes, and smoking status. Overlap of identified DMRs with placental DNA methylation will be studied as described previously. Identified differences in methylation profiles in cfDNA could–at least partly–originate from methylation differences in the placenta [22]. Moreover, total cfDNA including maternal-originated cfDNA could also make an important contribution to biomarker development, as demonstrated by others for preeclampsia prediction [21]. Depending on the identification of disease-specific DMRs, we will develop a disease-prediction tool based on top-ranked DMRs for each trimester. DMRs will be used to generate receiver operating characteristic (ROC) curves based on the reference samples, this will allow us to score individual samples on a specific threshold per DMR. Samples having methylation scores above the threshold will score a 1, while samples below the threshold score a 0, leading to a cumulative methylation score per individual sample. In sub-study 3, we aim to validate the first trimester disease-associated DMRs identified in sub-study 1 in an independent cohort using residual NIPT samples. Cases and controls will additionally be matched for gestational age at NIPT sampling.

Subsequently, pathway analyses of genes containing identified DMRs will be performed using http://geneontology.org.

## Dissemination

Findings from this study will be disseminated by publication of peer-reviewed manuscripts and presentations at scientific conferences. All study data leading to publications will be available upon reasonable request. Raw sequencing data (fastq files) will be deposited on the Sequence Read Archive (SRA) database. Participants of the Predict study will be informed about the progress and results of this study through a yearly newsletter.

## Research implications

The placenta is a crucial but largely inaccessible organ during development, and plays an important role in the pathophysiology of several obstetric complications. Unravelling the epigenetic programming of the placenta already during pregnancy in health and disease could be of great interest to increase our understanding of how epigenetic mechanisms influence placental and fetal health and development.

We are the first study longitudinally investigating cfDNA methylation dynamics during gestation using MeD-seq, a technology with an extensive genome-wide coverage. Identified DNA methylation changes can provide more insight in normal placental development. Estimating gestational age using the cfDNA methylome with a clinically relevant precision would require the identification of methylation profiles specific for a small gestational age window (e.g. 1–2 weeks), which would also require more sampling moments throughout gestation. However, this study could serve as a first proof of concept by the identification of trimester-specific methylation profiles in cfDNA. Additionally, placental DNA methylation differences have been associated–either as cause, consequence or both–with several obstetric complications with a major burden for fetal and maternal health and implications for health outcomes across the life course. For example, several studies investigating postpartum placentas from pregnancies complicated by preeclampsia have shown DNA methylation differences in genomic regions relevant to the pathophysiology of preeclampsia, such as angiogenesis and cell proliferation [7]. Identified disease-specific DMRs in our study could allow for further unravelling of underlying (epigenetic) mechanisms contributing to the development of associated diseases at different time points in pregnancy. Moreover, identified DMRs may be used as biomarker for (early) prediction of disease. Early detection of high-risk pregnancies is key to improve health outcomes for both mother and child, by enabling timely initiation of adequate treatment and prevention [24–26].

Although genome-wide analyses of the cfDNA methylome during pregnancy is largely understudied, cfDNA holds great promise to study epigenetic (placental) programming during pregnancy and to develop clinically relevant biomarkers for obstetrical care. Our study could therefore form a basis for future studies into cfDNA applications with the ultimate aim to improve prenatal and future health for mother and child.

## Supporting information

S1 File SPIRIT checklist. (DOC)

S2 File Study protocols of described sub-studies. (PDF)

S1 Fig Number of identified DMRs between cfDNA from pregnant women in the first trimester, as compared to cfDNA from non-pregnant controls with increasing sample size (preliminary data). The number of identified DMRs stabilizes after including 8 pregnant and 8 non-pregnant cfDNA samples (8v8).
(TIF)

## References

[1] Moore L, Le T, Fan G. DNA methylation and its basic function. Neuropsychopharmacology. 2012;38(1):23–38. doi: 10.1038/npp.2012.112 PubMed Central PMCID: PMC3521964. 22781841 PMC3521964

[2] Januar V, Desoye G, Novakovic B, Cvitic S, Saffery R. Epigenetic regulation of human placental function and pregnancy outcome: considerations for causal inference. Am J Obstet Gynecol. 2015;213(4 Suppl):S182–96. doi: 10.1016/j.ajog.2015.07.011 26428498

[3] Nelissen E, Montfoort van A, Dumoulin J, Evers J. Epigenetics and the placenta. Hum Reprod Update. 2011;17(3):397–417. doi: 10.1093/humupd/dmq052 20959349

[4] Novakovic B, Yuen R, Gordon L, Penaherrera M, Sharkey A, Moffett A, et al. Evidence for widespread changes in promoter methylation profile in human placenta in response to increasing gestational age and environmental/stochastic factors. BMC Genomics. 2011;12:529. PubMed Central PMCID: PMC3216976. doi: 10.1186/1471-2164-12-529 22032438 PMC3216976

[5] Yuan V, Hui D, Yin Y, Peñaherrera M, Beristain A, Robinson W. Cell-specific characterization of the placental methylome. BMC Genomics. 2021;6;22(1):6. PubMed Central PMCID: PMC7788826. doi: 10.1186/s12864-020-07186-6 33407091 PMC7788826

[6] Zhang B, Kim M, Elliot G, Zhou Y, Zhao G, Li D, et al. Human placental cytotrophoblast epigenome dynamics over gestation and alterations in placental disease. Dev Cell. 2021;56(9):1238–1252.e5. PubMed Central PMCID: PMC8650129. doi: 10.1016/j.devcel.2021.04.001 33891899 PMC8650129

[7] Cruz de OJ, Conceição I, Tosatti J, Gomes K, Luizon M. Global DNA methylation in placental tissues from pregnant with preeclampsia: A systematic review and pathway analysis. Placenta. 2020;101:97–107. doi: 10.1016/j.placenta.2020.09.004 32942147

[8] Cirkovic A, Garovic V, Lazovic J, Milicevic O, Savic M, Rajovic N, et al. Systematic review supports the role of DNA methylation in the pathophysiology of preeclampsia: a call for analytical and methodological standardization. Biol Sex Differ. 2020;6;11(1):36. doi: 10.1186/s13293-020-00313-8 PubMed Central PMCID: PMC7336649. 32631423 PMC7336649

[9] Wilson S, Robinson W. Utility of DNA methylation to assess placental health. Placenta. 2018;64 Suppl 1:S23–S28. doi: 10.1016/j.placenta.2017.12.013 29273273

[10] Toure D, Elrayes W, Barnes-Josiah D, Hartman T, Klinkebiel D, Baccaglini L. Epigenetic modifications of human placenta associated with preterm birth: a systematic review. J Matern Fetal Neonatal Med. 2018;31(4):530–541. doi: 10.1080/14767058.2017.1291620 28282769

[11] Wang X, Tian F, Fan L, Xie C, Niu Z, Chen W. Comparison of DNA methylation profiles associated with spontaneous preterm birth in placenta and cord blood. BMC Med Genomics. 2019;12(1):1. doi: 10.1186/s12920-018-0466-3 PubMed Central PMCID: PMC6318854. 30606219 PMC6318854

[12] Deshpande S, Balasinor N. Placental Defects: An Epigenetic Perspective. Reprod Sci. 2018;25(8):1143–1160. doi: 10.1177/1933719118766265 29642799

[13] Bianco-Miotto T, Mayne B, Buckberry S, Breen J, Rodriquez Lopez C, Roberts C. Recent progress towards understanding the role of DNA methylation in human placental development. Reproduction. 2016;152(1):R23–30. PubMed Central PMCID: PMC PMC5064761. doi: 10.1530/REP-16-0014 27026712 PMC5064761

[14] Banister C, Koestler D, Maccani M, Padbury J, Houseman E, Marsit C. Infant growth restriction is associated with distinct patterns of DNA methylation in human placentas. Epigenetics. 2011;6(7):920–7. doi: 10.4161/epi.6.7.16079 PubMed Central PMCID: PMC3154432. 21758004 PMC3154432

[15] Herzog E, Eggink A, Willemsen S, Slieker R, Wijnands K, Felix J, et al. Early- and late-onset preeclampsia and the tissue-specific epigenome of the placenta and newborn. Placenta. 2017;Oct;58:122–132. doi: 10.1016/j.placenta.2017.08.070 28962690

[16] Apicella C, Ruano C, Mehats C, Miralles F, Vaiman D. The Role of Epigenetics in Placental Development and the Etiology of Preeclampsia. Int J Mol Sci. 2019;Jun 11;20(11):2837. doi: 10.3390/ijms20112837 31212604 PMC6600551

[17] Ashraf U, Hall D, Rawls A, Alexander B. Epigenetic processes during preeclampsia and effects on fetal development and chronic health. Clin Sci (Lond). 2021;135(19):2307–2327. PubMed Central PMCID: PMC8948502. doi: 10.1042/CS20190070 34643675 PMC8948502

[18] Hu H, Liu H, Peng C, Deng T, Fu X, Chung C, et al. Clinical experience of non-invasive prenatal chromosomal aneuploidy testing in 190,277 patient samples. Curr Mol Med. 2016;(16:759–66.). doi: 10.2174/1566524016666161013142335 27739366

[19] Wong A, Lo Y. Noninvasive fetal genomic, methylomic, and transcriptomic analyses using maternal plasma and clinical implications. Trends Mol Med. 2015;21(2):98–108. doi: 10.1016/j.molmed.2014.12.006 25618775

[20] Hui W, Chiu R. Noninvasive prenatal testing beyond genomic analysis: what the future holds. Curr Opin Obstet Gynecol. 2016;Apr;28(2):105–10. doi: 10.1097/GCO.0000000000000252 26866842

[21] Spinelli M, Zdanowicz J, Keller I, Nicholson P, Raio L, Amylidi-Mohr S, et al. Hypertensive disorders of pregnancy share common cfDNA methylation profiles. Sci Rep 2022;Nov 18;12(1):19837. doi: 10.1038/s41598-022-24348-6; PubMed Central PMCID: PMC9674847. 36400896 PMC9674847

[22] De Borre M, Che H, Yu Q, Lannoo L, De Ridder K, Vancoillie L, et al. Cell-free DNA methylome analysis for early preeclampsia prediction. Nat Med. 2023;Sep;29(9):2206–2215. doi: 10.1038/s41591-023-02510-5 37640858

[23] He W, Zhang Y, Wu K, Wang Y, Zhao X, Lv L, et al. Epigenetic phenotype of plasma cell-free DNA in the prediction of early-onset preeclampsia. J Obstet Gynaecol. 2023;Dec;43(2):2282100. doi: 10.1080/01443615.2023.2282100 38038254

[24] Poon L, McIntyre H, Hyett J, Borges da Fonseca E, Hod M. The first-trimester of pregnancy—A window of opportunity for prediction and prevention of pregnancy complications and future life. Diabetes Res Clin Pract. 2018;Nov;145:20–30. doi: 10.1016/j.diabres.2018.05.002 29852233

[25] Dimitriadis E, Rolnik D, Zhou W, Estrada-Gutierrez G, Koga K, Francisco R, et al. Pre-eclampsia. Nat Rev Dis Primers. 2023 Feb 16;9(1):8. doi: 10.1038/s41572-023-00417-6 36797292

[26] Roberge S, Bujold E, Nicolaides K. Aspirin for the prevention of preterm and term preeclampsia: systematic review and metaanalysis. Am J Obstet Gynecol. 2018;Mar;218(3):287–293.e1. doi: 10.1016/j.ajog.2017.11.561 29138036

[27] Lee Y, Choufani S, Weksberg R, Wilson S, Yuan V, Burt A, et al. Placental epigenetic clocks: estimating gestational age using placental DNA methylation levels. Aging (Albany NY). 2019;11(12):4238–4253. doi: 10.18632/aging.102049 PubMed Central PMCID: PMC6628997. 31235674 PMC6628997

[28] Jiang P, Tong Y, Sun K, Cheng S, Leung T, Chan K, et al. Gestational Age Assessment by Methylation and Size Profiling of Maternal Plasma DNA: A Feasibility Study. Clin Chem. 2017;63(2):606–608. doi: 10.1373/clinchem.2016.265702 27979959

[29] Butt K, Lim K. Determination of gestational age by ultrasound. J Obstet Gynaecol Can. 2014;36(2):171–181. doi: 10.1016/S1701-2163(15)30664-2 24518917

[30] Moss J, Magenheim J, Neiman D, Zemmour H, Loyfer N, Korach A, et al. Comprehensive human cell-type methylation atlas reveals origins of circulating cell-free DNA in health and disease. Nat Commun. 2018;Nov 29;9(1):5068. doi: 10.1038/s41467-018-07466-6 PubMed Central PMCID: PMC6265251. 30498206 PMC6265251

[31] Hestand M, Bessem M, van Rijn P, de Menezes R, Sie D, Bakker I, et al. Fetal fraction evaluation in non-invasive prenatal screening (NIPS). Eur J Hum Genet. 2019;Feb;27(2):198–202. doi: 10.1038/s41431-018-0271-7 PubMed Central PMCID: PMC6336813. 30254213 PMC6336813

[32] Deng C, Liu S. Factors Affecting the Fetal Fraction in Noninvasive Prenatal Screening: A Review. Front Pediatr. 2022;Jan 27;10:812781. doi: 10.3389/fped.2022.812781 PubMed Central PMCID: PMC8829468. 35155308 PMC8829468

[33] Grunau C, Clark S, Rosenthal A. Bisulfite genomic sequencing: systematic investigation of critical experimental parameters. Nucleic Acids Res. 2001;Jul 1;29(13):E65–5. doi: 10.1093/nar/29.13.e65 PubMed Central PMCID: PMC55789. 11433041 PMC55789

[34] Wreczycka K, Gosdschan A, Yusuf D, Grüning B, Assenov Y, Akalin A. Strategies for analyzing bisulfite sequencing data. J Biotechnol. 2017;261(105–115). doi: 10.1016/j.jbiotec.2017.08.007 28822795

[35] Li Y. Modern epigenetics methods in biological research. Methods. 2021 Mar;187:104–113. doi: 10.1016/j.ymeth.2020.06.022 PubMed Central PMCID: PMC7785612. 32645449 PMC7785612

[36] Giannini L, Boers R, van der Ende E, Poos J, Jiskoot L, Boers J, et al. Distinctive cell-free DNA methylation characterizes presymptomatic genetic frontotemporal dementia. Ann Clin Transl Neurol. 2024;Mar;11(3):744–756. doi: 10.1002/acn3.51997 PubMed Central PMCID: PMC10963298. 38481040 PMC10963298

[37] Eikenboom E, Wilting S, Deger T, Srebniak M, Van Veghel-Plandsoen M, Boers R, et al. Liquid Biopsies for Colorectal Cancer and Advanced Adenoma Screening and Surveillance: What to Measure?. Cancers (Basel). 2023;Sep 17;15(18):4607. doi: 10.3390/cancers15184607 PubMed Central PMCID: PMC10526371. 37760576 PMC10526371

[38] Bos M, Verhoeff S, Oosting S, Menke-van der Houven van Oordt W, Boers R, Boers J, et al. Methylated Cell-Free DNA Sequencing (MeD-seq) of LpnPI Digested Fragments to Identify Early Progression in Metastatic Renal Cell Carcinoma Patients on Watchful Waiting. Cancers (Basel). 2023;Feb 21;15(5):1374. doi: 10.3390/cancers15051374 PubMed Central PMCID: PMC10000042. 36900167 PMC10000042

[39] Boers R, Boers J, Tan B, van Leeuwen M, Wassenaar E, Sanchez E, et al. Retrospective analysis of enhancer activity and transcriptome history. Nat Biotechnol. 2023;Nov;41(11):1582–1592. doi: 10.1038/s41587-023-01683-1 PubMed Central PMCID: PMC10635829. 36823354 PMC10635829

[40] Boers R, Boers J, Hoon de B, Kockx C, Ozgur Z, Molijn A, et al. Genome-wide DNA methylation profiling using the methylation-dependent restriction enzyme LpnPI. Genome Res. 2018;28(1):88–99. PubMed Central PMCID: PMC5749185. doi: 10.1101/gr.222885.117 29222086 PMC5749185

[41] Deger T, Boers R, de Weerd V, Angus L, van der Put M, Boers J, et al. High-throughput and affordable genome-wide methylation profiling of circulating cell-free DNA by methylated DNA sequencing (MeD-seq) of LpnPI digested fragments. Clinical Epigenetics. 2021;13(1). doi: 10.1186/s13148-021-01177-4 34670587 PMC8529776

[42] Rousian M, Schoenmakers S, Eggink A, Gootjes D, Koning A, Koster M, et al. Cohort Profile Update: the Rotterdam Periconceptional Cohort and embryonic and fetal measurements using 3D ultrasound and virtual reality techniques. Int J Epidemiol 2021. doi: 10.1093/ije/dyab030 34097026 PMC8580268

[43] Steegers-Theunissen R, Verheijden-Paulissen J, van Uitert E, Wildhagen M, Exalto N, Koning A, et al. Cohort Profile: The Rotterdam Periconceptional Cohort (Predict Study). Int J Epidemiol 2016;45(2):374–81. doi: 10.1093/ije/dyv147 26224071

[44] van der Meij K, Sistermans E, Macville M, Stevens S, Bax C, Bekker M, et al. TRIDENT-2: national implementation of genome-wide non-invasive prenatal testing as a first-tier screening test in the Netherlands. Am J Hum Genet. 2019;105:1091–1101. doi: 10.1016/j.ajhg.2019.10.005 PubMed Central PMCID: PMC6904791. 31708118 PMC6904791

[45] Van der Meij K, De Groot-van Mooren M, Carbo E, Pieters M, Rodenburg W, Sistermans E, et al. Uptake of fetal aneuploidy screening after the introduction of the non-invasive prenatal test: A national population-based register study. Acta Obstet Gynecol Scand. 2021;Jul;100(7):1265–1272. doi: 10.1111/aogs.14091 PubMed Central PMCID: PMC8359325. 33465829 PMC8359325

[46] Magee L, Brown M, Hall D, Gupte S, Hennessy A, Karumanchi S, et al. The 2021 International Society for the Study of Hypertension in Pregnancy classification, diagnosis & management recommendations for international practice. Pregnancy Hypertens. 2022;Mar;27:148–169. doi: 10.1016/j.preghy.2021.09.008 35066406

[47] Gormley M, Oliverio O, Kapidzic M, Ona K, Hall S, Fisher S. RNA profiling of laser microdissected human trophoblast subtypes at mid-gestation reveals a role for cannabinoid signaling in invasion. Development. 2021;Oct 15;148(20):dev199626. doi: 10.1242/dev.199626 PubMed Central PMCID: PMC8572005. 34557907 PMC8572005

[48] Boers J, Boers R, Sakoltchik J, Dasgupta S, Martens L, Tadema K, et al. DNA methylation database for gynecological cancer detection, classification and assay development. bioRxiv. 2024;07.01.601485. doi: 10.1101/2024.07.01.601485

