# Supplementary material for: Methylome profiling of cell-free DNA during the early life course in (un)complicated pregnancies using MeD-seq: Protocol for a cohort study embedded in the prospective Rotterdam periconception cohort
Source: PLoS One. 2025 Jan 9;20(1):e0310019. doi: 10.1371/journal.pone.0310019 (PMC11717202; doi:10.1371/journal.pone.0310019)
Supplement: S2 File — (PDF) [file pone.0310019.s002.pdf]

Supplemental file S2

Methylome profiling of cell free DNA during the early life course in (un)complicated pregnancies using MeD-seq: protocol for a cohort study embedded in the prospective Rotterdam periconception cohort

S2.1 Study protocol sub-study 1

S2.2 Study protocol sub-study 2

S2.3 Study protocol sub-study 3

S2.1 Study protocol sub-study 1

Addendum 3 - Identification of methylation profiles in cell free DNA in the context of pregnancy related diseases using MeD-seq

Introduction

DNA methylation is an epigenetic regulator and plays a vital role in placenta development and function.(1, 2) DNA methylation is known to change with increasing gestational age (3-5) and changes in placental DNA methylation are associated with multiple maternal, gestational and placental related complications, like pre-eclampsia, gestational diabetes mellitus, preterm birth and intra-uterine growth restriction (6-12). Therefore, the study of placental DNA methylation potentially provides more insight in normal placental development and underlying pathophysiology of pregnancy related diseases and can be used to develop both gestational clock-based methylation profiles as well as disease specific biomarkers.

Usually, obtaining placental tissue during pregnancy requires invasive procedures (e.g. chorionic villus sampling). However, cfDNA originated from placental trophoblasts can be found in maternal plasma providing the opportunity to study placental DNA methylation in a non-invasive manner. Currently, cfDNA is already widely used for prenatal testing of fetal aneuploides.(13) In addition, different studies have shown an increase in the level of (placenta-derived) cfDNA in pregnancies complicated by pre-eclampsia.(14-19) However, since cfDNA is derived from dying cells, multiple factors including acute exercise and obesity can have an impact on maternal cfDNA quantity affecting the placenta-derived fraction as well.(20, 21) Specificity of cfDNA as a biomarker for pregnancy related diseases could be improved by identifying disease-specific DNA methylation differences, ideally leading to biomarkers with the potential to identify women at risk of developing associated diseases at an early stage.

There are different methods to study genome-wide DNA methylation. Whole-genome bisulfite sequencing (WGBS) is able to reach >90% coverage of all cytosine-phosphate-guanine (CpG) sites in the human genome, but its use remains limited due to its high costs and technological challenges. Reduced representation bisulphite treatment or Illumina microarray technology are widely used, but are restricted to a limited subset of CpGs.(22) Therefore, the recently developed MeD-seq technique is a promising alternative. MeD-seq focusses CpG methylation analysis to only the methylated regions of the genome, therefore requiring less sequencing depth as compared to WGBS but allows for genome-wide methylation profiling of more than half of all potentially methylated CpGs.(23) Additionally, MeD-seq requires only a

limited amount of input DNA (24) making it a suitable technique to study methylation patterns using the relatively low levels of cfDNA available in clinical practice.

In summary, research gaps exist regarding cfDNA methylation changes related to gestational age in normal pregnancies, and how this is affected by different pregnancy related diseases. This is the first study to investigate gestational age and pregnancy related disease specific DNA methylation changes in cfDNA in all trimesters in a genome-wide manner using MeD-seq.

Primary Objective:

1. Identify pregnancy related disease specific methylation profiles in cfDNA in maternal plasma, with the potential to identify women at risk of developing pregnancy related complications including pre-eclampsia, intra-uterine growth restriction and pre-term birth.
2. Identify genes or genetic pathways involved in the development of maternal, gestational and placental related complications.

Secondary Objective: Identify trimester-specific methylation profiles (identified at 11 weeks, 22-24 weeks and 30-32 weeks of gestation and at time of delivery) in cfDNA in uncomplicated pregnancies to determine gestational age specific reference profiles and identify genes or genetic pathways involved in normal placental development.

The Rotterdam Periconceptie cohort (Predict studie)

The Predict study is a single center prospective longitudinal cohort study (Predict study, METC 2004-227) aiming to gain more insight in the development and underlying causes of fertility problems and pregnancy complications. The overall goal is to identify parameters able to predict the occurrence of these pathologies before or in the beginning of pregnancy, to ultimately prevent these complications.(25, 26)

Implementation

During thirty months, after approval of this addendum and informed consent from the participants, additional blood draws will be performed at the same day as the ultrasound visits for the Predict study around 11, 22-24 and 30-32 weeks of gestation and during delivery if the delivery takes place at the Erasmus MC. This blood draw at 11 weeks of gestation will be combined with the current blood draw in

first trimester which is already being performed within the Predict study. Two extra tubes of blood will be collected in CellSave tubes and processed into plasma to be stored at -80°C until isolation of cfDNA takes place.

Our primary outcome are differentially methylated regions (DMRs) found in these cfDNA methylation profiles between uncomplicated controls versus women who develop pregnancy related diseases like pre-eclampsia, intra-uterine growth restriction and pre-term birth. It is not possible to make a power calculation beforehand because it is not known what the methylation profiles will look like. However, based on previous experiments to generate reference profiles using MeD-seq, the number of reliable DMRs stabilizes when having a sample size of ±8-9 samples. The incidence of the above mentioned pregnancy related diseases in Europa are 4-5% for pre-eclampsia (27), 9% for preterm birth (28) and 8-10% for intra-uterine growth restriction (29). We aim to include 10 pre-eclampsia cases and account for 20-25% loss to follow up, including loss because of miscarriages. Therefore we intend to include 300 women in total. Because of the higher incidence of preterm birth and intra-uterine growth restriction it is likely this cohort will comprise at least 10 women affected by these complications as well.

We will identify specific DMRs in cfDNA from maternal plasma determined using MeD-seq and perform pathway analyses of genes associated with identified DMRs involved in associated diseases using http://geneontology.org in collaborations with the department of Developmental Biology, prof J.Gribnau, Erasmus MC.

References

1. Januar V DG, Novakovic B, Cvitic S, Saffery R. Epigenetic regulation of human placental function and pregnancy outcome: considerations for causal inference. Am J Obstet Gynecol. 2015;213(4 Suppl):S182-96.
2. Nelissen ECM MvA, Dumoulin JCM, Evers JLH. Epigenetics and the placenta. Hum Reprod Update. 2011;17(3):397-417.
3. Novakovic B YR, Gordon L, Penaherrera MS, Sharkey A, Moffett A, Craig JM, Robinson WP, Saffery R. Evidence for widespread changes in promoter methylation profile in human placenta in response to increasing gestational age and environmental/stochastic factors. BMC Genomics. 2011;12:529.
4. Yuan V HD, Yin Y, Peñaherrera MS, Beristain AG, Robinson WP. Cell-specific characterization of the placental methylome. BMC Genomics. 2021;6;22(1):6.
5. Zhang B KM, Elliot G, Zhou Y, Zhao G, Li D, Lowdon RF, Gormley M, Kapidzic M, Robinson JF, McMaster MT, Hong C, Mazor T, Hamilton E, Sears RL, Pehrsson EC, Marra MA, Jones SJM, Bilenky M, Hirst M, Wang T, Costello JF, Fisher SJ. Human placental cytotrophoblast epigenome dynamics over gestation and alterations in placental disease. Dev Cell. 2021;56(9):1238-1252.e5.
6. Cruz de O J CI, Tosatti JAG, Gomes KB, Luizon MR. Global DNA methylation in placental tissues from pregnant with preeclampsia: A systematic review and pathway analysis. Placenta. 2020;101:97-107.
7. Cirkovic A GV, Lazovic JM, Milicevic O, Savic M, Rajovic N, Aleksic N, Weissgerber T, Stefanovic A, Stanisavljevic D, Milic N. Systematic review supports the role of DNA methylation in the pathophysiology of preeclampsia: a call for analytical and methodological standardization. Biol Sex Differ. 2020;6;11(1):36.
8. Wilson SL RW. Utility of DNA methylation to assess placental health. Placenta. 2018;64 Suppl 1:S23-S28.
9. Toure DM EW, Barnes-Josiah D, Hartman T, Klinkebiel D, Baccaglini L. Epigenetic modifications of human placenta associated with preterm birth: a systematic review. J Matern Fetal Neonatal Med. 2018;31(4):530-541.
10. Wang X TF, Fan L, Xie C, Niu Z, Chen W. Comparison of DNA methylation profiles associated with spontaneous preterm birth in placenta and cord blood. BMC Med Genomics. 2019;12(1):1.
11. Banister CE KD, Maccani MA, Padbury JF, Houseman EA, Marsit CJ. Infant growth restriction is associated with distinct patterns of DNA methylation in human placentas. Epigenetics. 2011;6(7):920-7.
12. Lizárraga D G-GA. The Placenta as a Target of Epigenetic Alterations in Women with Gestational Diabetes Mellitus and Potential Implications for the Offspring. Epigenomes. 2021;May 10;5(2):13.
13. Wong AIC LD. Noninvasive fetal genomic, methylomic, and transcriptomic analyses using maternal plasma and clinical implications. Trends Mol Med. 2015;21(2):98-108.
14. Wu Y WA, Cheng W, Lanes A, Wen SW, Walker M. Association between Levels of Total Cell-Free DNA and Development of Preeclampsia-A Literature Review. AJP Rep. 2021;11(1):e38-e48.
15. Kolarova TR GH, Nelson JL, Lockwood CM, Shree R. At Preeclampsia Diagnosis, Total Cell-Free DNA Concentration is Elevated and Correlates With Disease Severity. J Am Heart Assoc. 2021;3;10(15):e021477.
16. Amaral LM SV, Kutcher ME, Spradley FT, Cavalli RC, Tanus-Santos JE, Palei AC. Circulating Total Cell-Free DNA Levels Are Increased in Hypertensive Disorders of Pregnancy and Associated with Prohypertensive Factors and Adverse Clinical Outcomes. . Int J Mol Sci. 2021;8;22(2):564.
17. Sarzynska-Nowacka U KP, Wielgos M. Is there a future for cell-free fetal dna tests in screening for preeclampsia? . Ginekol Pol. 2019;90(1):55-60.
18. Martin A KI, Badell M, Samuel A. Can the quantity of cell-free fetal DNA predict preeclampsia: a systematic review. . Prenat Diagn. 2014;34(7):685-91.

19. Contro E BD, Farina A. Cell-Free Fetal DNA for the Prediction of Pre-Eclampsia at the First and Second Trimesters: A Systematic Review and Meta-Analysis. Mol Diagn Ther. 2017;21(2):125-135.
20. Yuwono NL WK, Ford CE. The influence of biological and lifestyle factors on circulating cell-free DNA in blood plasma. eLife. 2021;Nov 9;10:e69679.
21. Fibke A GS, Caron A, Starks E, Parker JDK, Swanson L, Jouan L, Langlois S, Rouleau G, Rousseau F, Karsan A. Effect of preexamination conditions in a centralized-testing model of non-invasive prenatal screening. Clin Chem Lab Med. 2021;Nov 11;60(2):183-190.
22. Wreczycka K GA, Yusuf D, Grüning B, Assenov Y, Akalin A. Strategies for analyzing bisulfite sequencing data. J Biotechnol. 2017;261(105-115).
23. Boers R BJ, Hoon de B, Kockx C, Ozgur Z, Molijn A, IJcken van W, Laven J, Gribnau J. Genome-wide DNA methylation profiling using the methylation-dependent restriction enzyme LpnPI. Genome Res. 2018;28(1):88-99.
24. Deger T BR, de Weerd V, Angus L, van der Put MMJ, Boers JB, Azmani Z, van IJcken WFJ, Grünhagen DJ, van Dessel LF, Lolkema MPJK, Verhoef C, Sleijfer S, Martens JWM, Gribnau J, Wilting SM. High-throughput and affordable genome-wide methylation profiling of circulating cell-free DNA by methylated DNA sequencing (MeD-seq) of LpnPI digested fragments. Clinical Epigenetics. 2021;13(1).
25. Rousian M SS, Eggink AJ, Gootjes DV, Koning AHJ, Koster MPH, et al. . Cohort Profile Update: the Rotterdam Periconceptional Cohort and embryonic and fetal measurements using 3D ultrasound and virtual reality techniques. Int J Epidemiol 2021.
26. Steegers-Theunissen RP V-PJ, van Uitert EM, Wildhagen MF, Exalto N, Koning AH, et al. Cohort Profile: The Rotterdam Periconceptional Cohort (Predict Study). Int J Epidemiol 2016;45(2):374-81.
27. Abalos E CC, Grosso AL, Chou D, Say L. Global and regional estimates of preeclampsia and eclampsia: a systematic review. Eur J Obstet Gynecol Reprod Biol. 2013;Sep;170(1): 1-7.
28. Chawanpaiboon S VJ, Moller A, Lumbiganon P, Petzold M, Hogan D, Landoulsi S, Jampathong N, Kongwattanakul K, Laopaiboon M, Lewis C, Rattanakanokchai S, Teng DN, Thinkhamrop J, Watananirun K, Zhang J, Zhou W, Gülmezoglu AM. Global, regional, and national estimates of levels of preterm birth in 2014: a systematic review and modelling analysis. Lancet Glob Health. 2019;Jan;7(1):e37-e46.
29. Alkalay AL GJJ, Promerance JJ. Evaluation of neonates born with intrauterine growth retardation: review and practice guidelines. J Perinatol. 1998;Mar-Apr (18(2):142-51).

S2.2 Study protocol sub-study 2

The identification of placental DNA methylation patterns related to gestational age using MeD-seq

(17-02-2023)

**Full title of protocol**	The identification of placental DNA methylation patterns related to gestational age using MeD-seq
**Short title or Acronym**	Placental Methylation
**Version**	1.1
**Date**	17-02-2023
**Coordinating investigator/ project leader**	Not applicable
**Principal investigator(s)**	dr. S. Schoenmakers<br>Prof. dr. J. Gribnau
**Head of department of Obstetrics & Gynaecology**<br>**Head of Department of Developmental Biology**	Prof. dr. E.A.P. Steegers<br>Prof. dr. J. Gribnau
**Sponsor**	Erasmus MC<br>Department of Obstetrics and Gynaecology<br>Department of Developmental Biology
**Subsidising party**	Not applicable

TABLE OF CONTENTS

List of abbreviations and relevant definitions **4**

Protocol summary **5**

1. Introduction and rationale **7**

2. Objective(s) **8**

3. Study type **8**

4. Study population **9**

5. Methods **11**

6. Unexpected discoveries **12**

7. Exchange, sharing or transfer of data and/or human material and/or images outside the Erasmus MC **12**

8. Statistical analysis **13**

9. Recruitment and consent **13**

10. Handling and storage of data and images **14**

11. Handling and storage of human material **15**

12. Amendments **16**

13. Publication **16**

14. References **17**

15. Attachments **18**

LIST OF ABBREVIATIONS AND RELEVANT DEFINITIONS

**cfDNA**	Cell free DNA
**CpG**	Cytosine-phosphate-Guanine
**DMR**	Differentially Methylated Region
**DOHaD**	Developmental origins of health and disease
**DTA**	Data Transfer Agreement
**Exception consent**	Form Care for data Template , in Dutch: Formulier uitzondering toestemming
**FDR**	False Discovery Rate
**IC**	Informed Consent
**MeD-seq**	Methylated DNA sequencing
**MTA**	Material Transfer Agreement
**NWTC**	Non-WMO Review Committee; in Dutch: Niet WMO Toetsingscommissie
**UAVG**	Dutch Act on Implementation of the General Data Protection Regulation; in Dutch: Uitvoeringswet Algemene Verordening Gegevensbescherming
**WMO**	Medical Research Involving Human Subjects Act, in Dutch: Wet Medisch-wetenschappelijk Onderzoek met Mensen

PROTOCOL SUMMARY

DNA methylation is essential for normal placental development and function. Previous studies have shown differences in placental DNA methylation related to gestational age and in placenta-related diseases. Unfortunately, placental methylation profiles early and mid-gestation are largely understudied since obtaining placental tissue during pregnancy is invasive. A promising alternative to noninvasively study placental DNA methylation is placental-originated cell free DNA (cfDNA) in the maternal circulation, which is currently widely applied for non-invasive prenatal testing (NIPT). However, specificity of cfDNA to study placental DNA is hampered by the presence of circulating (so called background) maternal cfDNA.

In this study, we aim to identify differentially methylated regions (DMRs) in placental tissues between the three different trimesters of pregnancy using the recently developed Methylated DNA Sequencing (MeD-seq) technology. MeD-seq allows the study of DNA methylation genome-wide and provides a more extensive coverage of the genome compared to most other techniques. Importantly, it is compatible with low concentrations of circulating cfDNA. After identification of trimester-specific DMRs, the next step will involve analyses of underlying genes or genetic pathways related to these DMRs. Insight into dynamics of placental methylation and involved genes throughout pregnancy could increase our understanding of normal placental development. Moreover, a reference database of longitudinal placental methylation patterns of (uncomplicated) pregnancies based on direct placental tissues could serve as a blue-print to help distinguish the placental derived cfDNA fraction from the background maternal cfDNA in future studies, improving specificity of specific placental cfDNA as a non-invasive placental marker.

Placental samples will either be obtained via the Fetal Biobank Amsterdam (retrospectively), abortion clinics, including but potentially not limited to Gynaikon Kliniek Rotterdam and Bea & Bloemenhovekliniek (prospectively), and the Erasmus MC (both retrospectively and prospectively). The majority of first and second trimester samples from the Fetal Biobank Amsterdam and the Erasmus MC originate from pregnancies complicated by congenital defects or obstetric complications such as immature birth. Since, placental samples of uncomplicated pregnancies are needed as reference, first and second trimester samples from uncomplicated pregnancies terminated for social indications will be collected at abortion clinics. Third trimester placentas of both complicated and uncomplicated pregnancies will be collected at the Erasmus MC. DNA will be isolated from placental biopsies and subsequently be sequenced using MeD-seq. Based on previous experiments generating reference profiles from different tissues using MeD-seq, we aim to include a minimum of 10 samples per trimester both retrospectively and prospectively.

Participants retrospectively recruited either already provided informed consent to use placental tissue for scientific research, or these women made no objection to the use of residual material at the time of their outpatient clinic appointment during pregnancy. For prospectively collected placental tissues obtained via an abortion clinic, informed consent will be asked by an independent nurse or medical doctor working at the abortion clinic after opting for termination of the pregnancy. These tissues will be collected anonymously and would otherwise be discarded after the procedure. For participants prospectively

recruited in first, second or third trimester at the Erasmus MC, informed consent will be asked by a member of the research group and tissues will be coded before use. Since only residual tissues will be obtained and used, participants will not undergo any additional procedures.

1. Introduction and rationale

The developmental origins of health and disease (DOHaD) hypothesis is supported by accumulating evidence showing the importance of the gestational environment for fetal development as well as for health outcomes later in life (1-3). Epigenetic mechanisms, of which DNA methylation is the most studied, have been proposed to play a role in affecting developmental programming (1-3) and are essential for normal placental development and function (4, 5). Impaired placentation has been associated with multiple adverse pregnancy outcomes. We and others have shown differentially methylated regions (DMRs) in postpartum placentas in pregnancies affected by a range of obstetric complications, including preeclampsia, intra-uterine growth restriction and preterm birth. This indicates a role for epigenetic mechanisms in disease development, either as cause or consequence. (6-14)

Placental methylation profiles early and mid-gestation are largely understudied due to the impossibility to non-invasively investigate placental tissue during pregnancy. Previous studies using tissues from elective abortions showed that placental DNA methylation in general increases with advanced gestational age (15-17). A promising alternative to study placental DNA methylation noninvasively already during pregnancy is by use of so called cell free DNA (cfDNA) originating from turnover and spill of placental cell types into the maternal circulation. Currently, cfDNA is already widely used for non-invasive prenatal testing for chromosomal aneuploidies as part of prenatal screening.(18) However, on average only 13% (range 3-30%) of total cfDNA within the maternal circulation originates from the placenta (19), with the remaining cfDNA originating from maternal tissues, hampering specificity of selection and detection of placental DNA methylation.

From the different methods to study genome-wide DNA methylation we will use the recently developed MeD-seq technology which focusses on regional changes in DNA methylation, requiring less sequencing depth as compared to whole-genome bisulfite sequencing while providing a more extensive coverage of the genome as compared to most other techniques.(20, 21) Besides, MeD-seq requires a limited amount of input DNA and is therefore one of the few technologies compatible with the low cfDNA levels available in clinical practice.(22)

Since no placental DNA methylation studies have yet been performed using MeD-seq, no suitable (technique-specific) reference methylation profiles per trimester exist. Here, we aim to identify trimester-specific DMRs in placental tissues in uncomplicated and complicated pregnancies using MeD-seq. Second, we intend to identify underlying genes or genetic pathways related to identified DMRs, hereby increasing our understanding of normal placental development. Moreover, identified placental DNA methylation profiles could serve as a blue-print to subsequently help distinguish the placental derived fraction from the maternal one in total cfDNA in the maternal circulation during pregnancy, improving specificity of cfDNA as placental marker in future studies into cfDNA using MeD-seq (Figure 1). This could lead to numerous applications, including the identification of disease-specific DNA methylation changes with the potential to be used as novel noninvasive biomarkers for early prediction of placenta-related diseases in future studies.

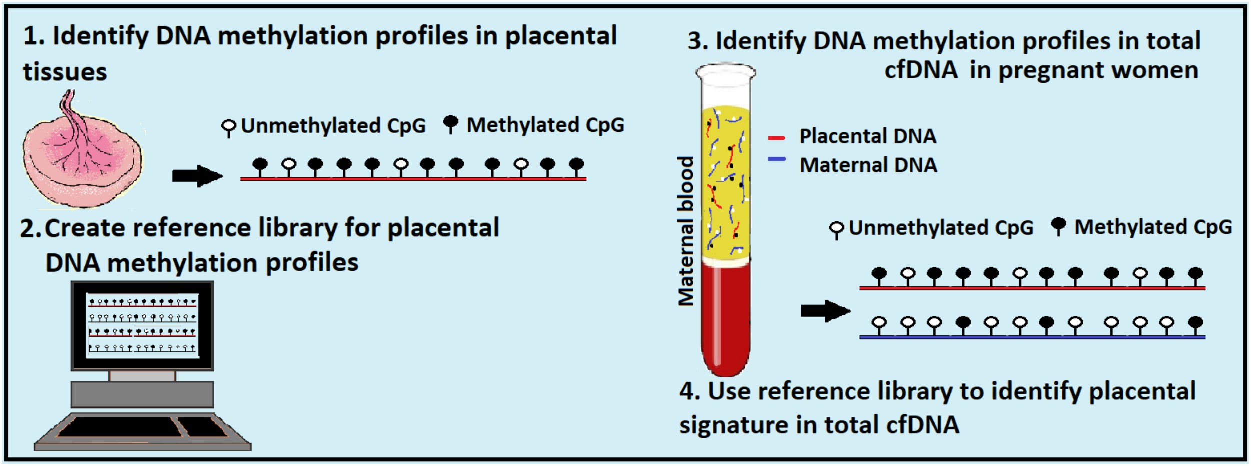

Figure 1: Schematic overview of how a reference database of placental methylation patterns based on direct placental tissues could serve as a blue-print to help distinguish the placental derived fraction in total maternal cfDNA in future studies. CpG = cytosine-phosphate-guanine site; cfDNA = cell free DNA

2. Objective(s)

Primary objective:

1) Identify trimester-specific DMRs in placental tissues of uncomplicated and complicated pregnancies using MeD-seq;

Secondary objectives:

2) Identify underlying genes or genetic pathways related to identified DMRs involved in (ab)normal placental development.
3) Identify placental DNA methylation profiles to specifically distinguish the placental derived fraction from the maternal one in total cfDNA in the maternal circulation during pregnancy in future studies using MeD-seq.

3. Study type

3.1 Study type:

☐ Retrospective
☐ Prospective
☒ Combination Retrospective/Prospective

3.2 Check all the applicable boxes:

- [x] Medical records (re-use of data from healthcare, including AI)
- [ ] Case report
- [ ] Re-use data from research
- [ ] Evaluations of quality of healthcare (retrospective)
- [x] Research with additional use of residual material from regular healthcare
- [x] Research with re-use of human materials from research or existing biobank
- [ ] Research with human materials without biobank
- [ ] De novo biobank (human material obtained without burdensome or invasive procedures)
- [ ] Post marketing survey research with medical devices
- [ ] Phase IV research
- [ ] Healthcare evaluation research (prospective)
- [ ] Medical devices
- [ ] In Vitro Diagnostic Tests
- [ ] Other research, *describe*

4. Study population

4.1 Population (base)

Placental samples will either be obtained via the Fetal Biobank Amsterdam (retrospectively), abortion clinics, including but potentially not limited to Gynaikon Kliniek Rotterdam and Bea & Bloemenhovekliniek (prospectively), and the Erasmus MC (both retrospectively and prospectively). The majority of first and second trimester samples from the Fetal Biobank Amsterdam and the Erasmus MC originate from pregnancies complicated by congenital defects or obstetric complications such as immature birth. Since, placental samples of uncomplicated pregnancies are needed as reference, first and second trimester samples from uncomplicated pregnancies terminated for social indications will be collected at abortion clinics. Third trimester placentas of both complicated and uncomplicated pregnancies will be collected at the Erasmus MC.

4.2 Inclusion criteria

In order to be eligible to participate in this study, a subject must meet all of the following criteria:

- For samples retrospectively collected: residual placental tissue available at the Fetal Biobank Amsterdam or Erasmus MC from singleton pregnancy;

- For samples prospectively collected at the Erasmus MC or abortion clinic:
    - Participant has to be at least 18 years of age
    - Singleton pregnancy
    - Participant is willing and able to give written informed consent
    - Additional for abortion clinic: voluntarily chosen for elective, surgical abortion for social indication, before potential participation in this study is discussed.

4.3 Exclusion criteria

Potential participants undergoing an abortion at the abortion clinic will be excluded from study participation if they are infected with HIV, hepatitis B or other blood borne infectious diseases. This is an exclusion criteria set by the abortion clinic for participation in any research with residual tissues for security reasons. Additionally, samples retrospectively collected at the Erasmus MC for which no explicit informed consent is available, will be excluded if they have not seen a (research) nurse during their pregnancy to discuss the potential use of residual tissue or when there is known objection against use of material for research purposes in the Erasmus MC ‘database bezwaren’.

4.4 Sample size calculation

Our primary outcomes are DMRs in placental DNA between different trimesters. Since no placental methylation studies have been performed using MeD-seq, it is unknown what the methylation profiles will look like. Power calculations are therefore not possible. Based on previous experiments to generate reference profiles using MeD-seq in other tissues, the number of reliable DMRs stabilizes when having a sample size of a minimum of 10 samples. Therefore, we aim to include at least 10 different samples per trimester both retrospectively and prospectively.

4.5 (Planned) start date

We plan to start right after METC approval.

4.6 (Planned) end data

We expect to finish within 1.5 years after the start of this study.

5. Methods

5.1 *Please describe how you do conduct research and which methods are used, e.g. questionnaires, human material collection, extraction of data from medical records, analysis of images, the use of an app/medical device/diagnostic test, clinical tests to be performed, etc. Include information on frequency, duration, volumes, if a Data Management Plan is available (then please upload DMP).*

Placenta samples from all three trimesters from both complicated and uncomplicated pregnancies will be obtained in multiple ways as described earlier: via the Fetal Biobank Amsterdam (retrospectively), abortion clinics (prospectively) and the Erasmus MC (both retrospectively and prospectively). After sample collection, DNA will be isolated from placental biopsies. MeD-seq technology will be used to investigate DNA methylation profiles between different trimesters in both complicated and uncomplicated pregnancies. Custom Python scripts will be used to process the acquired DNA methylation profiles. Raw data files (fastq) will be filtered to be able to distinguish methylation data from background data and will be mapped to the human genome (hg38) using bowtie. To subsequently analyse DMRs, we will generate both regional (promoter, gene body and cytosine-phosphate-guanine (CpG) Island regions) as genome-wide methylation scores. For the genome-wide analysis, a sliding window technique will be used to detect DMRs. The Chi-squared test will be used for statistical testing with either the False Discovery Rate (FDR) or Bonferroni correction for multiple testing. A FDR or Bonferroni adjusted p-value <0.05 will be considered significant. After DMRs are determined, Z-score transformation of the read count data will be applied for unsupervised hierarchical clustering analysis. Pathway analyses will be performed using http://geneontology.org.

5.2 Standard clinical care versus extra for research:
Indicate which of the methods are part of standard clinical care and which tests and/or visits are extra for research purposes i.e. not standard clinical care.

Since only residual tissues will be used, participants will not undergo any additional procedures. The placentas would otherwise be discarded. Sample collection and MeD-seq is only for research purposes.

5.3 *Please describe the burden and risks associated with participation, e.g. the amount and number of blood samples, biopsies, liquor, hair, urine, nails, saliva etc., the number of site visits, physical examinations or other tests, questionnaires or diaries that have to be filled out, physical and psychological discomfort associated with participation.*

Not applicable since participants will not undergo any additional procedures.

5.4 *A risk-benefit analysis must be given, if applicable.*
If a non-therapeutic study is carried out with minors or incapacitated subjects, it should be specified whether the risks are negligible and the burden minimal and why the study is group related

(i.e., study can only be done using these patients groups).

The participants will not personally benefit from the study. However, the obtained information can lead to a better understanding of placental development in normal pregnancies. In the future, our aim is to use this information to be able to understand normal placental development over the course of pregnancy, unravel underlying pathophysiology of placenta-related complications and to develop biomarkers to predict disease risks at an early stage of pregnancy. Participants will not undergo any additional risks related to this study.

5.5 Medical device(s)/In vitro diagnostic tests:
Describe which medical device and/or in vitro diagnostic test is used, what the intended use of the medical device is and whether the medical device/diagnostic test is already available in the Erasmus MC.

Not applicable.

6. Unexpected discoveries

6.1 Is there a chance of unexpected discoveries?

☐ Yes

☒ No

6.2 *If yes, describe the procedures, who will be notified, how the subjects are notified.*

7. Exchange, sharing or transfer of data and/or human material and/or images outside Erasmus MC

Describe with which organization the data and/or human material and/or images are shared, are they profit or non-profit organizations, whether these organizations are in the EU or outside the EU, how the privacy of subjects is protected outside the Erasmus MC and describe the procedures regarding the exchange(s), whether a Data Transfer Agreement/Material Transfer Agreement is available (if yes, please upload the DTA/MTA).

We will obtain placental samples via different Dutch abortion clinics and from the Fetal Biobank Amsterdam. All are non-profit organizations in the Netherlands. This data is only shared with us anonymously so subjects privacy is protected. We will not share the samples we obtained from the Fetal Biobank Amsterdam. For samples prospectively collected at an abortion clinic, informed consent to share data and/or placental tissues with third parties is asked in the informed consent form. However, at this moment there are no plans to share these tissues with other organizations outside the Erasmus MC.

8. Statistical analysis

8.1 Main study parameter/endpoint:

Trimester-specific DMRs in placental tissues of uncomplicated and complicated pregnancies using MeD-seq.

8.2 Secondary study parameters/endpoints:

Underlying genes or genetic pathways related to identified DMRs involved in (ab)normal placental development.

8.3 Other study parameters:

Identified placental DNA methylation profiles could contribute to specifically distinguish the placental derived fraction from the maternal one in total cfDNA in the maternal circulation during pregnancy in future studies using MeD-seq.

9. Recruitment and consent

9.1 Will the subjects be asked for informed consent?

☐ Yes *(Upload Patient Information Letter and Informed Consent)*

☐ No, only anonymous data is used, i.e. the data can never be traced back to an individual subject

☐ No, this research will be performed under the exception consent
(Upload form Care for data Template, in Dutch: Formulier uitzondering toestemming)

☒ Other (e.g. partly, indirectly) *Please describe the situation.*

For the retrospective analyses:

First and second trimester participants who will be recruited at the Fetal Biobank Amsterdam and third trimester participants who will be recruited at the Erasmus MC already provided informed consent for use of their residual placental material for (future) research (IC forms are attached).

First and second trimester participants who will be recruited at the Erasmus MC have not provided informed consent. However, since these pregnancies were mostly terminated because of congenital birth defects or spontaneous immature deliveries, it is emotionally undesirable to ask these patients in retrospect for informed consent to use residual placental material for this specific study. As part of regular care, pregnant women who visit the outpatient clinic of the obstetrics department have an appointment with a (research) nurse who, among others, discusses the potential use of residual material for research purposes. An information letter is also send to these women (see attached). Using an ‘opting-out’ system, only objection

against the use of residual material is registered in the patient record and in the ‘database bezwaren’. We will limit the use of placental samples to women who have had an appointment with a (research) nurse during their pregnancy. Potential participants with known objection against material use for research according to the Erasmus MC “database bezwaren” will be excluded.

<u>For the prospective analyses</u>:

For participants who will be recruited via an abortion clinic (first or second trimester) or at the Erasmus MC (first, second, or third trimester), informed consent will be asked before sample collection as described below. Patient information letters and informed consent forms are attached.

9.2 *If yes, please give a description of the recruitment and informed consent procedures. How and by whom (investigator, supervising doctor, other person) participants will be informed about the study and asked for their consent, how much time will they be given to consider the decision. The patient information letter with informed consent form should be attached as a separate document.*

Women enrolled at an abortion clinic will be informed about the possibility to participate in this study on a voluntary base after deciding for termination of the pregnancy. Study information will be given by an independent nurse or medical doctor working at the abortion clinic who is not involved in the study, as well as by the informed consent form. After informing about the study and asking informed consent, a potential participant can consider participating in this study until the surgical abortion takes place. Patient information letter and informed consent form are attached.

Women prospectively enrolled at the Erasmus MC in the first / second / third trimester will be informed about the possibility to participate in this study by a member of the research team and via the informed consent form. A potential participant can (re)consider participating in this study until the placenta is born. Patient information letter and informed consent form are attached.

9.3 If no, exception consent: *describe how it is safeguarded that subjects are excluded who have objected against the re-use of their data, human material, images.*

Potential participants recruited retrospectively at the Erasmus MC with known objection against material use for research according to the Erasmus MC “database bezwaren” will be excluded.

10. Handling and storage of data and images

10.1 *Describe how subject’s privacy is protected. Describe how, when and by whom data is coded, and how the key table is safeguarded.*

Samples from the abortion clinic and samples from the Fetal Biobank are shared with us anonymously and therefore privacy of these participants is guaranteed. Placental samples obtained at the Erasmus MC will be coded by the researcher before DNA isolation from samples and data-analysis will be performed. The key to the study-ID's is kept separately and remains on site with the local research team.

10.2 *Describe how data is stored (i.e. which data management system/data capture system), who has access to the coded source data, how long data will be kept, which steps are taken to ensure data security, what happens with the data after the research has been completed.*

Data will be stored at secured servers at the Erasmus MC. Study documents and tissue samples are stored for 15 years after the end of the study. The only individually identifiable information collected is patient's ID for samples collected at the Erasmus MC and this information is kept separately from the research data and is only accessible to the research team, the study monitor, and Inspectie Gezondheidszorg en Jeugd (IGJ). The handling of personal data will comply with the EU General Data Protection Regulation and the Dutch Act on Implementation of the General Data Protection Regulation (in Dutch: Algemene Verordening Gegevensbescherming en Uitvoeringswet Algemene Verordening Gegevensbescherming).

10.3 *Describe how images are stored, how the subject's privacy is protected, what happens with images after the research has been completed.*

Not applicable

10.4 *Describe how approval of re-use of data and/or images is obtained.*

No (additional) approval will be obtained from participants who already provided informed consent or for samples retrospectively collected at the Erasmus MC. For women recruited prospectively at an abortion clinic or at the Erasmus MC, approval of re-use of data will be asked via the IC form.

11. Handling and storage of human material

11.1 *Please describe which human material is used.*

Placental tissues collected from different trimesters.

11.2 Check all the boxes which are applicable to the human material origin:

☒ Regular clinical care

☒ Research

☒ Left-over human material

☐ De Novo biobank material
☐ Other, *please specify*

11.3 How is human material handled and stored?

☒ Anonymous, i.e. the material can never be traced back to an individual subject

➔ This is the case for samples collected at the Fetal Biobank Amsterdam and abortion clinics

☒ Pseudonymised/Coded

➔ This is the case for samples collected at the Erasmus MC

☐ Identifiable

11.4 *In case of new human material collection, describe how the human material is coded and stored (which registration system, Central Biobank or other location), who has access to the registration system and human material, by whom the key to the code is safeguarded, how long and where human material will be kept, what happens with the human material after the research has been completed.*

Newly collected placental samples from the abortion clinic will be shared with us anonymously and will be given a random study ID. Newly collected samples at the Erasmus MC will also be coded before storage. The key to the code remains on site with the local research team. Newly collected tissues will be stored at the local research department for 15 years. The research team has access to the registration system and materials. After 15 years the material will be destroyed.

11.5 *Describe how approval for re-use of human material is obtained.*

No (additional) approval will be obtained from participants who already provided informed consent or for samples retrospectively collected at the Erasmus MC. For women prospectively recruited at an abortion clinic or at the Erasmus MC, approval of re-use of their materials will be asked via the IC form.

12. Amendments

Amendments are changes made to the research after a favourable opinion by the NWTC has been given. All amendments must be submitted to the NWTC that gave the favourable opinion. Substantial amendments must be approved by the NWTC before they can be implemented

13. Publication

Do you have the intention to submit the study results in a manuscript for publication in a journal:

☒ Yes
☐ No, *please motivate*

14. References

1. Ashraf UM HD, Rawls AZ, Alexander BT. Epigenetic processes during preeclampsia and effects on fetal development and chronic health. Clin Sci (Lond). 2021;135(19):2307-2327.
2. Peral-Sanchez I HB, Ojeda DA, Steegers-Theunissen RPM, Willaime-Morawek S. Epigenetics in the Uterine Environment: How Maternal Diet and ART May Influence the Epigenome in the Offspring with Long-Term Health Consequences. Genes (Basel). 2021;13(1):31.
3. Gluckman P.D HM, Cooper, C, Thornburg, K. Effect of in utero and early-life conditions on adult health and disease. N Engl J Med. 2008;359(1):61-73.
4. Januar V DG, Novakovic B, Cvitic S, Saffery R. Epigenetic regulation of human placental function and pregnancy outcome: considerations for causal inference. Am J Obstet Gynecol. 2015;213(4 Suppl):S182-96.
5. Nelissen ECM MvA, Dumoulin JCM, Evers JLH. Epigenetics and the placenta. Hum Reprod Update. 2011;17(3):397-417.
6. Cruz de O J CI, Tosatti JAG, Gomes KB, Luizon MR. Global DNA methylation in placental tissues from pregnant with preeclampsia: A systematic review and pathway analysis. Placenta. 2020;101:97-107.
7. Cirkovic A GV, Lazovic JM, Milicevic O, Savic M, Rajovic N, Aleksic N, Weissgerber T, Stefanovic A, Stanisavljevic D, Milic N. Systematic review supports the role of DNA methylation in the pathophysiology of preeclampsia: a call for analytical and methodological standardization. Biol Sex Differ. 2020;6;11(1):36.
8. Wilson SL RW. Utility of DNA methylation to assess placental health. Placenta. 2018;64 Suppl 1:S23-S28.
9. Toure DM EW, Barnes-Josiah D, Hartman T, Klinkebiel D, Baccaglini L. Epigenetic modifications of human placenta associated with preterm birth: a systematic review. J Matern Fetal Neonatal Med. 2018;31(4):530-541.
10. Wang X TF, Fan L, Xie C, Niu Z, Chen W. Comparison of DNA methylation profiles associated with spontaneous preterm birth in placenta and cord blood. BMC Med Genomics. 2019;12(1):1.
11. Deshpande SS BN. Placental Defects: An Epigenetic Perspective. Reprod Sci. 2018;25(8):1143-1160.
12. Bianco-Miotto T MB, Buckberry S, Breen J, Rodriquez Lopez CM, Roberts CT. Recent progress towards understanding the role of DNA methylation in human placental development. Reproduction. 2016;152(1):R23-30.
13. Banister CE KD, Maccani MA, Padbury JF, Houseman EA, Marsit CJ. Infant growth restriction is associated with distinct patterns of DNA methylation in human placentas. Epigenetics. 2011;6(7):920-7.
14. Herzog EM EA, Willemsen SP, RC Slieker, Wijnands KPJ, Felix JF, Chen J, Stubbs A, Van der Spek PJ, Van Meur JB, Steegers-Theunissen RPM. Early- and late-onset preeclampsia and the tissue-specific epigenome of the placenta and newborn. Placenta. 2017;Oct;58:122-132.
15. Novakovic B YR, Gordon L, Penaherrera MS, Sharkey A, Moffett A, Craig JM, Robinson WP, Saffery R. Evidence for widespread changes in promoter methylation profile in human placenta in response to increasing gestational age and environmental/stochastic factors. BMC Genomics. 2011;12:529.

16. Yuan V HD, Yin Y, Peñaherrera MS, Beristain AG, Robinson WP. Cell-specific characterization of the placental methylome. BMC Genomics. 2021;6;22(1):6.
17. Zhang B KM, Elliot G, Zhou Y, Zhao G, Li D, Lowdon RF, Gormley M, Kapidzic M, Robinson JF, McMaster MT, Hong C, Mazor T, Hamilton E, Sears RL, Pehrsson EC, Marra MA, Jones SJM, Bilenky M, Hirst M, Wang T, Costello JF, Fisher SJ. Human placental cytotrophoblast epigenome dynamics over gestation and alterations in placental disease. Dev Cell. 2021;56(9):1238-1252.e5.
18. Wong AIC LD. Noninvasive fetal genomic, methylomic, and transcriptomic analyses using maternal plasma and clinical implications. Trends Mol Med. 2015;21(2):98-108.
19. Hu H LH, Peng C, Deng T, Fu X, Chung C, Zhang E, Lu C, Zhang K, Liang Z, Yang Y. . Clinical experience of non-invasive prenatal chromosomal aneuploidy testing in 190,277 patient samples. Curr Mol Med. 2016(16:759–66.).
20. Boers R BJ, Hoon de B, Kockx C, Ozgur Z, Molijn A, IJcken van W, Laven J, Gribnau J. Genome-wide DNA methylation profiling using the methylation-dependent restriction enzyme LpnPI. Genome Res. 2018;28(1):88-99.
21. Wreczycka K GA, Yusuf D, Grüning B, Assenov Y, Akalin A. Strategies for analyzing bisulfite sequencing data. J Biotechnol. 2017;261(105-115).
22. Deger T BR, de Weerd V, Angus L, van der Put MMJ, Boers JB, Azmani Z, van IJcken WFJ, Grünhagen DJ, van Dessel LF, Lolkema MPJK, Verhoef C, Sleijfer S, Martens JWM, Gribnau J, Wilting SM. High-throughput and affordable genome-wide methylation profiling of circulating cell-free DNA by methylated DNA sequencing (MeD-seq) of LpnPI digested fragments. Clinical Epigenetics. 2021;13(1).

15. Attachments

Patients information letter

Information letter for participation in scientific research

Research into the programming and composition of healthy placentas

1. Introduction

Dear Madam,

We understand that this is a difficult moment to be approached with a request to participate in scientific research. We would like to emphasize that participation is completely voluntary.

You are receiving this information letter because:

- You are at least 18 years old
- You have an unwanted pregnancy and you choose a surgical termination of your pregnancy
- You do not have HIV/aids, Hepatitis B or any other contagious disease
- There are no known congenital abnormalities in the fetus

In this letter you can read what the research is about and what participation would mean for you.

Are you interested?

- Please read this information letter carefully.
- Ask questions to your healthcare provider who gives you this information.

If you want to participate, we ask you to fill out the consent form (Appendix B).

2. General Information

The study was set up by the Erasmus Medical Centre, Rotterdam. The research is carried out by researchers from the Department of Obstetrics & Gynecology in collaboration with the Department of Developmental Biology and your abortion clinic.

The Non-WMO Assessment Committee from the Erasmus MC has assessed whether this research falls within the scope of the Medical Research Involving Human Subjects Act (WMO) or not, and has assessed and approved the content of the research.

3. What is the purpose of this research?

The aim of this research is to gain more knowledge about the normal development of the placenta at different times in pregnancy. By gaining more insight into the course of this development in healthy pregnancies, we hope to use this knowledge to better understand what goes wrong in pregnancies with complications such as preeclampsia and fetal growth retardation. This knowledge will hopefully contribute to the prevention of these complications in the future.

4. What does participation mean for you?

You do not notice anything from your participation in this study. The study will not affect your treatment. After removal of the pregnancy tissue, a piece of the placenta is stored. The remaining tissue, including fetal tissue, is not used for this study. Participation in the survey is completely anonymous. When you participate in the study, the only information shared by your abortion clinic with the Erasmus MC is:
1. How many weeks pregnant you were at the time of pregnancy termination
No personal data is collected from you and participation is therefore anonymous.

5. What are the advantages and disadvantages of participating in the study?

You will not benefit (directly) from participating in this study. Your participation will contribute to more knowledge about the normal development of the placenta. There are no disadvantages for you with regard to additional actions or treatments.

6. If you do not want to participate or want to stop participating in the study

Participation in the research is entirely voluntary. Only if you agree to participate, you sign the consent form. You can withdraw your consent for the study as long as the tissue can still be traced back to you. This is the case as long as the tissue is still at the abortion clinic. If you later wish to withdraw your consent, this is no longer possible: we store all tissues anonymously, so we no longer know which tissue belonged to you. We therefore ask you to report this as soon as possible if you no longer wish to participate. You do not have to say *why* you are stopping.

7. What data do we collect?

(Medical) Data
The only data collected is the gestational age at the time of the termination.

Human Tissue
We collect a piece of the placenta that was removed during your abortion treatment.

Genetic Material
For this research we collect and process genetic material from the placenta. We are interested in changes in placental programming during pregnancy. The information is not used to study or store any (genetic) information of the parents.

8. What do we do with your data and human tissue?

Why do we collect, use and store your data and tissue?
We collect, use and store your data and tissue in order to answer the questions of this research. We want to publish the results of the study in a scientific journal so that other researchers and doctors can also learn from this information.

How do we protect your privacy?
The researchers will not know from who the tissues came. The study is conducted anonymously to protect your privacy. If you give permission, you will be asked to fill in a consent form. The placental tissues and this form cannot be traced back to each other.

How long do we store your data and tissues?
We store your data and tissues at the Erasmus MC for 15 years in order to use it for this research. After this we destroy your tissues.

May we use your data and/or body material for other research?
After this study, your data may also be important for other research into the development of the placenta. You can indicate on the consent form whether you agree with this. If you do not give permission for the use of your data in future research, you can still participate in this study.

We ask you separately on the consent form if you would agree with possible sending of your anonymous data and tissue material to other parties if they would like to use this data for research in the future. If you do not give this permission, you can still participate in this study.

Of course, if you give your approval, your data and tissue material will only be shared anonymously with other research institutes or parties for medical-scientific research. When data is provided to countries outside the European Union (so-called 'third countries'), such as the US, it is not possible to achieve exactly the same level of protection as within the EU. You can indicate separately on the consent form whether you consent to the provision of your anonymous data and tissue materials outside the European Union. If you do not give this permission, you can still participate in this study.

Would you like to know more about your privacy?
Would you like to know more about your rights with regard to the processing of personal data? Then look up https://www.autoriteitpersoonsgegevens.nl/nl/over-privacy/persoonsgegevens.
If you have any questions or complaints about your privacy, we recommend that you first discuss these with your care giver at your abortion clinic. You can also go to the Data Protection Officer of the Erasmus MC. Or you can submit a complaint to the Dutch Data Protection Authority.

9. Do you receive compensation for participating?

You will not be reimbursed for your participation and there are no additional costs involved.

10. Do you have any questions?

This research was assessed by the Erasmus MC Non-WMO Assessment Committee. According to this committee, this research does not fall under the Medical Research Involving Human Subjects Act. (Wet medisch-wetenschappelijk onderzoek met mensen)
If you have any questions about this study, please contact the researchers.
The contact details can be found in Appendix A.

Finally, we wish you strength during this period.

Yours sincerely,
The research team

Appendix A: Contact Information

Principal Investigator:
Dr. S. Schoenmakers, gyneacologist
E-mail. s.schoenmakers@erasmusmc.nl

Fellow investigator:
Drs. M.M. van Vliet, physician-researcher
E-mail: m.m.vanvliet@erasmusmc.nl

Complaints: The Erasmus MC Complaints Committee
Digital complaint form via www.erasmusmc.nl/nl-nl/patientenzorg/klachtenopvang-enklachtenbemiddeling
Phone number: 010-704 41 08

Data Protection Officer Erasmus MC:
The Data Protection Officer from the Erasmus MC can be reached via the secretariat of the Legal Affairs department.
E-mail: functionaris.gegevensbescherming@erasmusmc.nl
Phone: 010-703 4986

For more information or if you have any questions about your rights, please contact the Data Protection Officer or the Dutch Data Protection Authority.

Appendix B: Informed consent form participant

Research into the programming and composition of healthy placentas

- I have read the information letter. I was able to ask questions which have been answered sufficiently. I had enough time to decide whether I want to participate in this study.
- I know participation is voluntarily. I know I can stop participating as long as my tissues can be traced back to me. I do not have to give a reason if I want to stop participating.
- I agree with the collection and use of my medical information and tissue material in the way as described in the information letter for the purposes of this study.
- I agree to store my medical information and tissue material for 15 years after this study at the Erasmus MC.

We ask you to choose YES or NO in the following table. If you choose NO, you can still participate in this study:

I agree to the use of my medical information for other research, as described in the information letter.	Yes ☐	No ☐
I agree to store (residual) tissue material for other research purposes, as described in the information letter. The tissues are being stored till 15 years after the end of this study.	Yes ☐	No ☐
I agree to sharing my information –anonymously- for research purposes in the future with commercial parties or research institutes outside the EU where European guidelines for protection of personal information are not valid.	Yes ☐	No ☐
I agree to sharing my tissues -anonymously- for research purposes in the future with commercial parties or research institutes outside the EU where European guidelines for protection of personal information are not valid.	Yes ☐	No ☐

- I agree to use my placental tissues for this study.

My name is (participant):____

Signature: Date : __ / __ / __

The participant can get the information letter and a copy of the informed consent form.

I declare that I have fully informed this participant about this study. If during the study, information becomes known that could affect the participant's consent, I will inform her timely.

Study information was given by:

Name:____

Function:____

Signature (paraaf): Date: __ / __ / __

S2.3 Study protocol sub-study 3

Prediction of placental related complications by DNA methylation profiles in cell free DNA collected for non-invasive prenatal testing (NIPT) using MeD-seq

Version 2.0

September 29, 2023

**Full title of protocol**	Prediction of placental related complications by DNA methylation profiles in cell free DNA collected for non-invasive prenatal testing (NIPT) using MeD-seq
**Protocol ID**	OZBS72.22409
**Short title or Acronym**	Epi-NIPT
**Version**	2.0
**Date**	29-09-2023
**Coordinating investigator/ project leader**	Not applicable
**Principal investigator**	Prof. dr. R.P.M. Steegers-Theunissen
**Sub investigator**	Prof. dr. J. Gribnau
**Head of department of Obstetrics & Gynaecology**<br><br>**Head of Department of Developmental Biology**	Prof. dr. E.A.P. Steegers<br><br>Prof. dr. J. Gribnau
**Sponsor**	Erasmus MC<br>Department of Obstetrics and Gynaecology<br><br>Department of Developmental Biology
**Subsidising party**	Not applicable

TABLE OF CONTENTS

List of abbreviations and relevant definitions 4

Protocol summary 5

1. Introduction and rationale 6

2. Objective(s) 7

3. Study type 7

4. Study population 8

5. Methods 9

6. Unexpected discoveries 10

7. Exchange, sharing or transfer of data and/or human material and/or images outside the Erasmus MC 10

8. Statistical analysis 11

9. Recruitment and consent 11

10. Handling and storage of data and images 12

11. Handling and storage of human material 12

12. Amendments 13

13. Publication 13

14. References 14

15. Attachments 15

LIST OF ABBREVIATIONS AND RELEVANT DEFINITIONS*

**cfDNA**	Cell free DNA
**DMR**	Differentially Methylated Region
**Exception consent**	Form Care for data Template , in Dutch: Formulier uitzondering toestemming
**FDR**	False Discovery Rate
**IC**	Informed Consent
**MEC**	Medical Ethics Committee
**MeD-seq**	Methylated DNA sequencing
**NIPT**	Non-invasive prenatal testing
**NWTC**	Non-WMO Review Committee; in Dutch: Niet WMO Toetsingscommissie
**UAVG**	Dutch Act on Implementation of the General Data Protection Regulation; in Dutch: Uitvoeringswet Algemene Verordening Gegevensbescherming
**WMO**	Medical Research Involving Human Subjects Act, in Dutch: Wet Medisch-wetenschappelijk Onderzoek met Mensen

PROTOCOL SUMMARY

DNA methylation plays a vital role in placental development and methylation differences are associated with multiple placental related complications. Placental-originated cell free DNA (cfDNA) circulates in maternal plasma, providing the opportunity to non-invasively study placental DNA methylation during pregnancy. Up until recently, cfDNA was already widely used for non-invasive prenatal testing (NIPT) on a trial base (TRIDENT-2 study) and cfDNA screening is currently implemented in regular health care.

We aim to identify differentially methylated regions (DMRs) in cfDNA from residual NIPT samples between women who at a later gestational age developed preeclampsia (group 1), intrauterine fetal growth restriction (group 2) and spontaneous preterm birth (group 3) all as compared to uncomplicated pregnancies (group 4). Included women who delivered at the Erasmus MC and/or participated in The Rotterdam Periconception Cohort (PREDICT study, MEC-2004-227) will retrospectively be selected..

We will use the Methylated DNA sequencing (MeD-seq) technology to identify DMRs genome-wide. MeD-seq requires a limited amount of input DNA and is therefore compatible with the low levels of cfDNA available in clinical practice. Based on previous experiments to generate reference profiles using MeD-seq, the number of identified DMRs stabilizes when having samples from ten different individuals. Therefore, to prevent the identification of unreliable DMRs (related to genomic variation), we intend to include twenty patients per group (training and validation set), with a total of four groups in this pilot study.

Since suboptimal implantation and placentation in the beginning of pregnancy is involved in the pathophysiology of placental related complications, we hypothesize to identify disease-specific DMRs already present at the end of first trimester. NIPT sampling is usually performed between 11 and 13 weeks of gestation. Identified DMRs can potentially serve as biomarkers able to predict disease risk.

Second, we aim to identify underlying genes or genetic pathways related to identified DMRs involved in disease development, improving our understanding of underlying pathophysiology. This study could potentially lead to an expansion of current NIPT (Epi-NIPT), and initially will serve as a validation cohort for future studies into cfDNA.

There will be no additional burden or risks for participants, since our study focusses on material and data already collected (in both TRIDENT-2 as the Predict study).

1. Introduction and rationale

Impaired placenta development is involved in the causality of important obstetric complications, including preeclampsia and intrauterine growth restriction, resulting in increased maternal and fetal morbidity and mortality.(1, 2) DNA methylation is an important epigenetic mechanism involved in the (re)programming of gene expression and plays a vital role in placental development.(3-5)

Emerging evidence indicates a role for DNA methylation in the development of different placental related complications, either as cause or consequence. We and others have shown DNA methylation differences in postpartum placental tissues between pregnancies complicated by different placental related diseases, including preeclampsia, intra-uterine growth restriction and preterm birth as compared to controls.(6-13) For example, using the Illumina HumanMethylation450K beadchip technology Herzog *et al.* identified 869 differentially methylated positions in postpartum placental tissues between pregnancies diagnosed with preeclampsia <34 weeks of gestation compared to preterm birth controls.(12) DNA methylation differences associated with placental related complications could provide more insight in underlying pathophysiology. Moreover, disease-specific DNA methylation differences could potentially serve as biomarkers, ideally able to predict the risk of associated diseases at an early stage when targeted preventative interventions are most effective.(14)

Previous DNA methylation studies mainly focussed on postpartum placental tissues. (6-13) However, cfDNA originating from turnover of placental cell types circulates in maternal plasma providing the exciting opportunity to study placental DNA methylation in a non-invasive manner already during pregnancy.(15) cfDNA is currently already widely used for non-invasive prenatal testing of aneuploidies.(15) Between April 2017 and April 2023, all pregnant women in the Netherlands were offered NIPT on a trial basis (TRIDENT-2 study) and the NIPT uptake increased to over 40% in 2018.(16, 17) At the Erasmus MC, residual NIPT samples collected in the last two years are stored at the department of clinical genetics. Last month, promising results were reported by others who also used residual NIPT samples, indicative of cfDNA methylome profiling as promising tool for the prediction of early onset preeclampsia which could improve future obstetric care.(18)

The Rotterdam Periconception Cohort (PREDICT study, MEC-2004-227) is an ongoing cohort study longitudinally following women during pregnancy from the periconception period onwards. (19, 20) Moreover, a new subcohort is embedded in the Predict study in which longitudinally cfDNA will be collected to study DNA methylation profiles in relation to placental related complications. DNA methylation profiles related to preeclampsia, intrauterine growth restriction or spontaneous preterm birth identified in NIPT samples can serve as a validation cohort for the new prospective subcohort study.

From the different methods to study genome-wide DNA methylation, we will use MeD-seq technology. MeD-seq requires a limited amount of input DNA and is therefore one of the few technologies compatible with the low levels of cfDNA available in clinical practice.(21) Besides, MeD-seq analyses focusses on regional

changes in DNA methylation, requiring less sequencing depth as compared to whole-genome bisulfite sequencing while providing a more extensive coverage of the genome as widely used microarrays or reduced representation bisulphite treatment.(22, 23)

In summary, we aim to identify DMRs in cfDNA from residual NIPT samples between women who at a later gestational age developed preeclampsia, intrauterine growth restriction or spontaneous preterm birth as compared to uncomplicated pregnancies. We intend to identify underlying genes or genetic pathways related to identified DMRs, hereby increasing our understanding of underlying pathophysiology. Identified DMRs can potentially serve as biomarkers able to predict placental related disease risk early in pregnancy. In the future, this could potentially lead to an expansion of current NIPT (Epi-NIPT). Moreover, this study will serve as a validation cohort for our future prospective subcohort.

2. Objective(s)

1. Identify specific DMRs in first trimester residual NIPT samples related to adverse pregnancy outcome such as preeclampsia, intrauterine growth restriction and spontaneous preterm birth.
2. Identify underlying genes or genetic pathways related to identified DMRs, involved in disease development.
3. Create a validation cohort of DMRs in cfDNA for prospective subcohorts.

3. Study type

3.1 Study type:

☒ Retrospective
☐ Prospective
☐ Combination Retrospective/Prospective

3.2 Check all the applicable boxes:

☑ Medical records (re-use of data from healthcare, including AI)
☐ Case report
☑ Re-use data from research
☐ Evaluations of quality of healthcare (retrospective)
☐ Research with additional use of residual material from regular healthcare
☑ Research with re-use of human materials from research or existing biobank
☐ Research with human materials without biobank

- ☐ De novo biobank (human material obtained without burdensome or invasive procedures)
- ☐ Post marketing survey research with medical devices
- ☐ Phase IV research
- ☐ Healthcare evaluation research (prospective)
- ☐ Medical devices
- ☐ In Vitro Diagnostic Tests
- ☐ Other research, *describe*

4. Study population

4.1 Population (base)

Eligible women participated in the TRIDENT-2 study and during the same pregnancy participated in the Predict study and/or delivered at the Erasmus MC Rotterdam.

Women who developed preeclampsia, intrauterine growth restriction or spontaneous preterm birth will be included and compared to controls with an uncomplicated pregnancy. We aim to include samples from twenty women per group (total n=80).

4.2 Inclusion criteria

In order to be eligible to participate in this study, a subject must meet all of the following criteria:

- Participated in the Predict study *or* delivered at the Erasmus MC Rotterdam.
- Participated in the TRIDENT-2 study during the same pregnancy and agreed with the use of residual material for research purposes.
- At least 18 years of age at the time of participating in above mentioned studies.
- Developed preeclampsia (n=20) *or* intrauterine growth restriction (n=20) *or* delivered spontaneously preterm (n=20) *or* had an uncomplicated pregnancy (n=20).

4.3 Exclusion criteria

None.

4.4 Sample size calculation

A power calculation beforehand is not possible because it is unknown how many and which methylation profiles will be identified. However, based on previous experiments to generate reference profiles using MeD-seq, the number of reliable DMRs stabilizes with a sample size of minimal 10 individuals as a consequence of filtering out SNPs and other genomic variation that in <10 samples might still be picked up as DMR. Therefore, in this

pilot study we include twenty patients per group, ten as training dataset and 10 patients for internal validation.

4.5 (Planned) start date

We plan to start the collection of additional residual NIPT samples right after METC approval.

4.6 (Planned) end date

Sequencing of samples and data-analysis are performed in the following six months.

5. Methods

5.1 *Please describe how you do conduct research and which methods are used, e.g. questionnaires, human material collection, extraction of data from medical records, analysis of images, the use of an app/medical device/diagnostic test, clinical tests to be performed, etc. Include information on frequency, duration, volumes, if a Data Management Plan is available (then please upload DMP).*

This study will use data previously collected in the Predict study, medical records, and blood samples previously collected for NIPT in the TRIDENT-2 study. Participation in the TRIDENT-2 study will be checked for Predict participants and for eligible patients who had complicated deliveries at the Erasmus MC Rotterdam in the last two years in collaboration with the Clinical Genetics Department at the Erasmus MC. Cases and controls will be selected until twenty patients in each group are selected. The MeD-seq technology will be used to investigate DNA methylation profiles in cfDNA between women who developed preeclampsia, intrauterine growth restriction or spontaneous preterm birth compared to uncomplicated control pregnancies. Custom Python scripts will be used to process the acquired DNA methylation profiles. Raw data files (fastq) will be filtered to be able to distinguish methylation data from background data and will be mapped to the human genome (hg38) using bowtie. To subsequently analyse DMRs, we will generate both regional (promoter, gene body and CpG Island regions) as genome-wide methylation scores. For the genome wide analysis, a sliding window technique will be used to detect DMRs. The Chi-squared test will be used for statistical testing with either the False Discovery Rate (FDR) or Bonferroni correction for multiple testing. A FDR or Bonferroni adjusted p-value <0.05 will be considered significant. After DMRs are determined, Z-score transformation of the read count data will be applied for unsupervised hierarchical clustering analysis. Pathway analyses will be performed using http://geneontology.org.

5.2 Standard clinical care versus extra for research: *Indicate which of the methods are part of standard clinical care and which tests and/or visits are extra for research purposes i.e. not standard clinical care.*

The data previously collected and the blood samples obtained for NIPT were in addition or part of standard clinical care. MeD-seq analyses will be for research purposes only. No additional clinical data or blood samples will be collected for this study.

5.3 *Please describe the burden and risks associated with participation, e.g. the amount and number of blood samples, biopsies, liquor, hair, urine, nails, saliva etc., the number of site visits, physical examinations or other tests, questionnaires or diaries that have to be filled out, physical and psychological discomfort associated with participation.*
Not applicable.

5.4 *A risk-benefit analysis must be given, if applicable. If a non-therapeutic study is carried out with minors or incapacitated subjects, it should be specified whether the risks are negligible and the burden minimal and why the study is group related (i.e., study can only be done using these patients groups).*
Not applicable.

5.5 Medical device(s)/In vitro diagnostic tests: *Describe which medical device and/or in vitro diagnostic test is used, what the intended use of the medical device is and whether the medical device/diagnostic test is already available in the Erasmus MC.*
Not applicable.

6. Unexpected discoveries

6.1 Is there a chance of unexpected discoveries?

☐ Yes

☒ No

6.2 *If yes, describe the procedures, who will be notified, how the subjects are notified.*

7. Exchange, sharing or transfer of data and/or human material and/or images outside Erasmus MC

Describe with which organization the data and/or human material and/or images are shared, are they profit or non-profit organizations, whether these organizations are in the EU or outside the EU, how the privacy of subjects is protected outside the Erasmus MC and describe the procedures regarding the exchange(s), whether a Data Transfer Agreement/Material Transfer Agreement is available (if yes, please upload the DTA/MTA).

Residual NIPT samples are stored at the Clinical Genetics department of the Erasmus MC. Therefore, there will be no transfer of data or material outside Erasmus MC but only between the department of Clinical Genetics and the department of Obstetrics & Gynaecology. This is inevitable, to be able to check the availability of a (residual) NIPT sample and subsequently link the NIPT sample to the correct patients.

After the samples are linked to the correct participant using date of birth, name and due date of the pregnancy, samples will be coded.

8. Statistical analysis

8.1 Main study parameter/endpoint:

DMRs specific for preeclampsia, intrauterine growth restriction and spontaneous preterm birth and uncomplicated pregnancies in first trimester residual NIPT samples.

8.2 Secondary study parameters/endpoints:

Identify underlying genes or genetic pathways related to identified DMRs, involved in disease development.

8.3 Other study parameters:
Not applicable

9. Recruitment and consent

9.1 Will the subjects be asked for informed consent?

☐ Yes *(Upload Patient Information Letter and Informed Consent)*

☐ No, only anonymous data is used, i.e. the data can never be traced back to an individual subject

☐ No, this research will be performed under the exception consent
(Upload form Care for data Template, in Dutch: Formulier uitzondering toestemming)

☒ Other (e.g. partly, indirectly) *Please describe the situation.*

Participants for this study have already provided written informed consent for the TRIDENT-2 study. Only participants who explicitly gave informed consent for the use of residual study materials to be used for other research questions including research questions regarding pregnancy outcomes will be included.

9.2 *If yes, please give a description of the recruitment and informed consent procedures. How and by whom (investigator, supervising doctor, other person) participants will be informed about the study and asked for their consent, how much time will they be given to consider the decision. The patient information letter with informed consent form should be attached as a separate document.*
Not applicable.

9.3 If no, exception consent: *describe how it is safeguarded that subjects are excluded who have objected against the re-use of their data, human material, images.*
Not applicable.

10. Handling and storage of data and images

10.1 *Describe how subject's privacy is protected. Describe how, when and by whom data is coded, and how the key table is safeguarded.*

Immediately after the NIPT samples from the TRIDENT-2 study are obtained and linked to selected participants, the samples will be coded, stored and analyzed using a research-ID. The key to the study ID's code remains on site with the local research team.

10.2 *Describe how data is stored (i.e. which data management system/data capture system), who has access to the coded source data, how long data will be kept, which steps are taken to ensure data security, what happens with the data after the research has been completed.*

Coded data will be accessible for members of the (Predict) research team, the study monitor and the Health and Youth Care Inspectorate. The coded samples will be shared for sequencing and data-analysis with the Developmental Biology department at the Erasmus MC. Data will we stored at secured servers at the Erasmus MC for 15 years after the end of this study. Only coded data will be shared in publications.

10.3 *Describe how images are stored, how the subject's privacy is protected, what happens with images after the research has been completed.*

Not applicable.

10.4 *Describe how approval of re-use of data and/or images is obtained.*
Not applicable, approval is already provided in previously signed IC form.

The handling of personal data will comply with the EU General Data Protection Regulation and the Dutch Act on Implementation of the General Data Protection Regulation (in Dutch: Algemene Verordening Gegevensbescherming en Uitvoeringswet Algemene Verordening Gegevensbescherming).

11. Handling and storage of human material

11.1 *Please describe which human material is used.*
Residual plasma samples collected for the TRIDENT-2 study will be used.

11.2 Check all the boxes which are applicable to the human material origin:

☐ Regular clinical care
☒ Research (TRIDENT-2 study)

☐ Left-over human material

☐ De Novo biobank material

☐ Other, *please specify*

11.3 How is human material handled and stored?

☐ Anonymous, i.e. the material can never be traced back to an individual subject

☒ Pseudonymised/Coded

☒ Identifiable

We need to match clinical data to the right blood sample collected in the TRIDENT-2 study. This can't be achieved fully coded. After matching of the blood samples, coded research IDs will be used for further handling, storage and analyses.

11.4 *In case of new human material collection, describe how the human material is coded and stored (which registration system, Central Biobank or other location), who has access to the registration system and human material, by whom the key to the code is safeguarded, how long and where human material will be kept, what happens with the human material after the research has been completed.*

Not applicable.

11.5 *Describe how approval for re-use of human material is obtained.*
Not applicable, approval is already provided in previously signed IC form.

12. Amendments

Amendments are changes made to the research after a favourable opinion by the NWTC has been given. All amendments must be submitted to the NWTC that gave the favourable opinion. Substantial amendments must be approved by the NWTC before they can be implemented.

13. Publication

Do you have the intention to submit the study results in a manuscript for publication in a journal:

☒ Yes

☐ No, *please motivate*

14. References

1. Steegers EAP VDP, Duvekot JJ, Pijnenborg R. Pre-eclampsia. Lancet. 2010;Aug 21;376(9741):631-44.

2. Nardozza LM CA, Zamarian AC, Mazzola JB, Silva CP, Marçal VM, Lobo TF, Peixoto AB, Araujo Júnior E. Fetal growth restriction: current knowledge. Arch Gynecol Obstet. 2017 May; 295(5):1061-1077.
3. Moore LD LT, Fan G. DNA methylation and its basic function. Neuropsychopharmacology. 2012;38(1):23-38.
4. Januar V DG, Novakovic B, Cvitic S, Saffery R. Epigenetic regulation of human placental function and pregnancy outcome: considerations for causal inference. Am J Obstet Gynecol. 2015;213(4 Suppl):S182-96.
5. Nelissen ECM MvA, Dumoulin JCM, Evers JLH. Epigenetics and the placenta. Hum Reprod Update. 2011;17(3):397-417.
6. Cruz de O J CI, Tosatti JAG, Gomes KB, Luizon MR. Global DNA methylation in placental tissues from pregnant with preeclampsia: A systematic review and pathway analysis. Placenta. 2020;101:97-107.
7. Cirkovic A GV, Lazovic JM, Milicevic O, Savic M, Rajovic N, Aleksic N, Weissgerber T, Stefanovic A, Stanisavljevic D, Milic N. Systematic review supports the role of DNA methylation in the pathophysiology of preeclampsia: a call for analytical and methodological standardization. Biol Sex Differ. 2020;6;11(1):36.
8. Wilson SL RW. Utility of DNA methylation to assess placental health. Placenta. 2018;64 Suppl 1:S23-S28.
9. Banister CE KD, Maccani MA, Padbury JF, Houseman EA, Marsit CJ. Infant growth restriction is associated with distinct patterns of DNA methylation in human placentas. Epigenetics. 2011;6(7):920-7.
10. Apicella C RC, Mehats C, Miralles F, Vaiman D. The Role of Epigenetics in Placental Development and the Etiology of Preeclampsia. Int J Mol Sci. 2019;Jun 11;20(11):2837.
11. Ashraf UM HD, Rawls AZ, Alexander BT. Epigenetic processes during preeclampsia and effects on fetal development and chronic health. Clin Sci (Lond). 2021;135(19):2307-2327.
12. Herzog EM EA, Willemsen SP, RC Slieker, Wijnands KPJ, Felix JF, Chen J, Stubbs A, Van der Spek PJ, Van Meur JB, Steegers-Theunissen RPM. Early- and late-onset preeclampsia and the tissue-specific epigenome of the placenta and newborn. Placenta. 2017;Oct;58:122-132.
13. Toure DM EW, Barnes-Josiah D, Hartman T, Klinkebiel D, Baccaglini L. Epigenetic modifications of human placenta associated with preterm birth: a systematic review. J Matern Fetal Neonatal Med. 2018;31(4):530-541.
14. Poon LC MH, Hyett JA, Borges da Fonseca E, Hod M. The first-trimester of pregnancy - A window of opportunity for prediction and prevention of pregnancy complications and future life. Diabetes Res Clin Pract. 2018;Nov;145:20-30.
15. Wong AIC LD. Noninvasive fetal genomic, methylomic, and transcriptomic analyses using maternal plasma and clinical implications. Trends Mol Med. 2015;21(2):98-108.
16. van der Meij KRM SE, Macville MVE, et al. TRIDENT-2: national implementation of genome-wide non-invasive prenatal testing as a first-tier screening test in the Netherlands. Am J Hum Genet. 2019;105:1091-1101. .
17. Van der Meij KRM DG-vMM, Carbo EWS, Pieters MJ, Rodenburg W, Sistermans EA, Cornel MC, Henneman L, Dutch NIPT consortium. Uptake of fetal aneuploidy screening after the introduction of the non-invasive prenatal test: A national population-based register study. Acta Obstet Gynecol Scand. 2021;Jul;100(7):1265-1272.
18. De Borre M CH, Yu Q, Lannoo L, De Ridder K, Vancoillie L, Dreesen P, Van Den Ackerveken M, Aerden M, Galle E, Breckpot J, Van Keirsbilck J, Gyselaers W, Devriendt K, Vermeesch JR, Van Calsteren K, Thienpont B. Cell-free DNA methylome analysis for early preeclampsia prediction. . Nat Med. 2023;Sep;29(9):2206-2215.

19. Rousian M SS, Eggink AJ, Gootjes DV, Koning AHJ, Koster MPH, et al. . Cohort Profile Update: the Rotterdam Periconceptional Cohort and embryonic and fetal measurements using 3D ultrasound and virtual reality techniques. Int J Epidemiol 2021.
20. Steegers-Theunissen RP V-PJ, van Uitert EM, Wildhagen MF, Exalto N, Koning AH, et al. Cohort Profile: The Rotterdam Periconceptional Cohort (Predict Study). Int J Epidemiol 2016;45(2):374-81.
21. Deger T BR, de Weerd V, Angus L, van der Put MMJ, Boers JB, Azmani Z, van IJcken WFJ, Grünhagen DJ, van Dessel LF, Lolkema MPJK, Verhoef C, Sleijfer S, Martens JWM, Gribnau J, Wilting SM. High-throughput and affordable genome-wide methylation profiling of circulating cell-free DNA by methylated DNA sequencing (MeD-seq) of LpnPI digested fragments. Clinical Epigenetics. 2021;13(1).
22. Boers R BJ, Hoon de B, Kockx C, Ozgur Z, Molijn A, IJcken van W, Laven J, Gribnau J. Genome-wide DNA methylation profiling using the methylation-dependent restriction enzyme LpnPI. Genome Res. 2018;28(1):88-99.
23. Wreczycka K GA, Yusuf D, Grüning B, Assenov Y, Akalin A. Strategies for analyzing bisulfite sequencing data. J Biotechnol. 2017;261(105-115).
