# Supplementary figures and images for: Methylome profiling of cell-free DNA during the early life course in (un)complicated pregnancies using MeD-seq: Protocol for a cohort study embedded in the prospective Rotterdam periconception cohort
Source: PLoS One. 2025 Jan 9;20(1):e0310019. doi: 10.1371/journal.pone.0310019 (PMC11717202; doi:10.1371/journal.pone.0310019)

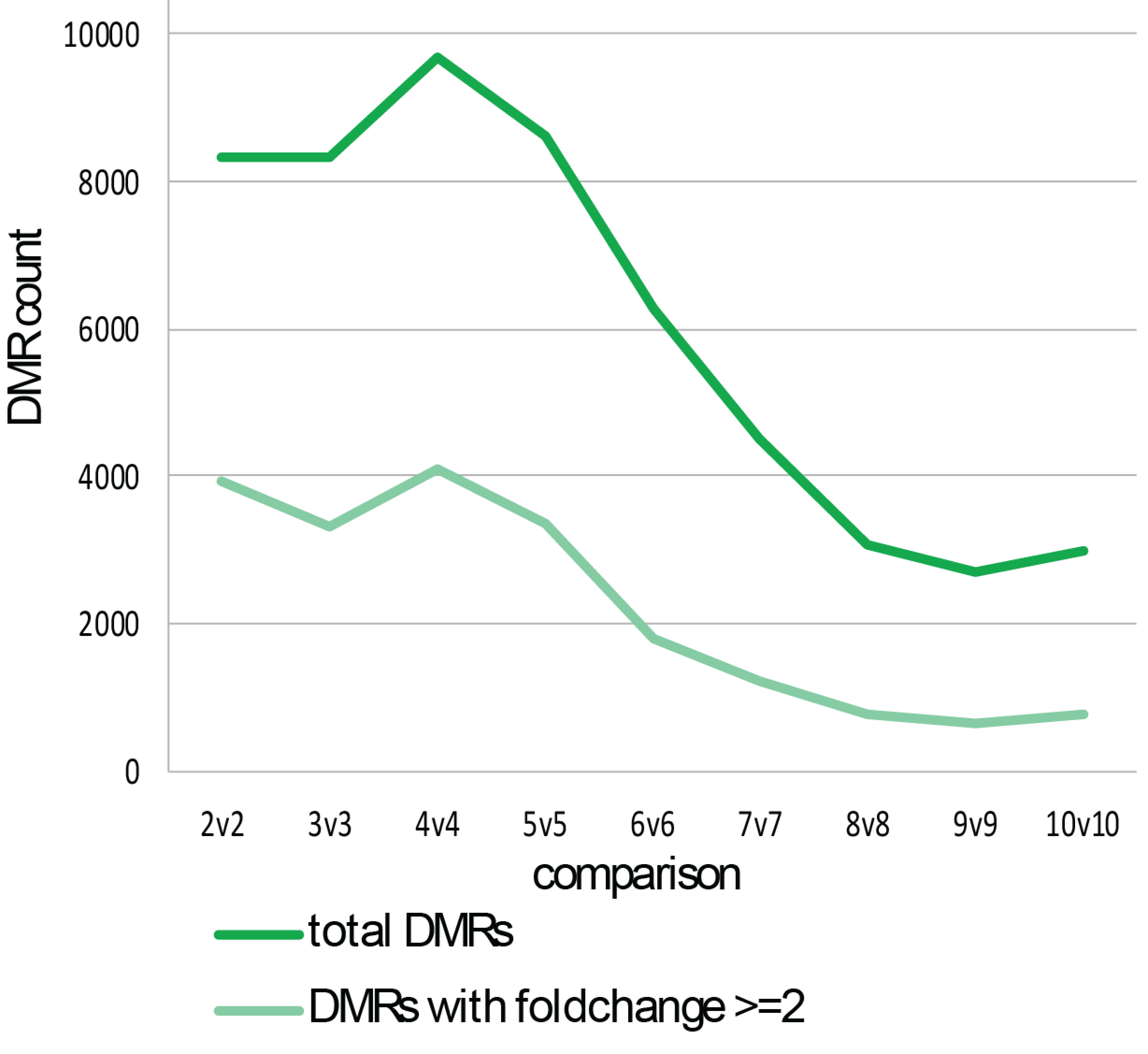

Supplement: S1 Fig — The number of identified DMRs stabilizes after including 8 pregnant and 8 non-pregnant cfDNA samples (8v8). (TIF) [file pone.0310019.s003.tif]
